# Distinct Evolutionary Origins of Intron Retention Splicing Events in *NHX1* Antiporter Transcripts Relate to Sequence Specific Distinctions in *Oryza* Species

**DOI:** 10.3389/fpls.2020.00267

**Published:** 2020-03-11

**Authors:** Gothandapani Sellamuthu, Vidya Jegadeeson, Radha Sivarajan Sajeevan, Raja Rajakani, Pavithra Parthasarathy, Kalaimani Raju, Lana Shabala, Zhong-Hua Chen, Meixue Zhou, Ramanathan Sowdhamini, Sergey Shabala, Gayatri Venkataraman

**Affiliations:** ^1^Plant Molecular Biology Laboratory, M.S. Swaminathan Research Foundation, Chennai, India; ^2^National Centre for Biological Sciences, Tata Institute of Fundamental Research, Bengaluru, India; ^3^Tasmanian Institute of Agriculture, College of Science and Engineering, University of Tasmania, Hobart, TAS, Australia; ^4^School of Science and Health, Hawkesbury Institute for the Environment, Western Sydney University, Penrith, NSW, Australia

**Keywords:** *Oryza*, *NHX1*, alternative splicing, intron retention, evolutionary origin

## Abstract

The genome of Asian cultivated rice (*Oryza sativa* L.) shows the presence of six organelle-specific and one plasma membrane (*OsNHX1-7*) NHX-type cation proton antiporters. Of these, vacuolar-localized *OsNHX1* is extensively characterized. The genus *Oryza* consists of 27 species and 11 genome-types, with cultivated rice, diploid *O. sativa*, having an AA-type genome. *Oryza NHX1* orthologous regions (gene organization, 5′ upstream cis elements, amino acid residues/motifs) from closely related *Oryza* AA genomes cluster distinctly from *NHX1* regions from more ancestral *Oryza* BB, FF and KKLL genomes. These sequence-specific distinctions also extend to two separate intron retention (IR) events involving *Oryza NHX1* transcripts that occur at the 5′ and 3′ ends of the NHX1 transcripts. We demonstrate that the IR event involving the 5′ UTR is present only in more recently evolved *Oryza* AA genomes while the IR event governing retention of the 13th intron of *Oryza NHX1* (terminal intron) is more ancient in origin, also occurring in halophytic wild rice, *Oryza coarctata* (KKLL). We also report presence of a retro-copy of the *OcNHX1* cDNA in the genome of *O. coarctata* (*rOcNHX1*). Preferential species and tissue specific up- or down-regulation of the correctly spliced *NHX1* transcript/5′ UTR/13th intron-retaining splice variants under salinity was observed. The implications of IR on *NHX1* mRNA stability and ORF diversity in *Oryza* spp. is discussed.

## Introduction

Plant growth, development and abiotic stress responses crucially depend on the regulation of intracellular ion homeostasis in different cellular endomembrane compartments (Bassil and Blumwald, 2014). To a large extent, cellular ion homeostasis is driven by compartment-specific activity of H^+^-translocating enzymes (H^+^-pumping ATPases or pyrophosphatases) that establish a H^+^ gradient utilized by other membrane transporters to couple the passive transport of H^+^ with an active uptake of ions (K^+^, Na^+^) against their electrochemical gradient (Martinoia et al., 2007; Amtmann and Leigh, 2009). This active uptake of ions provides the driving force vital for regulation of internal pH and cell volume expansion related processes (Bassil and Blumwald, 2014) as well as supporting metabolic activities of the cell. Na^+^/H^+^ exchangers (NHX) belong to monovalent cation/proton antiporter (CPA1) group of transporters (Maser et al., 2001; Sze and Chanroj, 2018). In plants, NHXs are ubiquitous, exist as multigene families and catalyze the electroneutral exchange of H^+^ for Na^+^ or K^+^. NHXs are present in both the plasma membrane (Shi et al., 2002) and in intracellular membranous compartments (IC), with the latter being localized to both vacuolar and endosomal membranes (Bassil and Blumwald, 2014). Previous research involving plant NHXs has primarily focused on their role in conferring salinity tolerance by Na^+^ sequestration in vacuoles under salinity. More recent studies suggest that, in addition to compartmentalizing Na^+^ in vacuoles in under salinity, they also have a role in maintaining K^+^ homeostasis (Bassil and Blumwald, 2014; Reguera et al., 2015; Bassil et al., 2019) and thus confer tissue salinity tolerance.

The genome of Asian cultivated rice, *Oryza sativa* L., shows the presence of one plasma membrane-based (*OsNHX7*/*OsSOS1*) and six organelle-specific (*OsNHX1-6*) antiporters. Of these, *OsNHX1* has been extensively characterized. The *OsNHX1* cDNA encodes a vacuolar antiporter (Fukuda et al., 1999), with expression of the *OsNHX1* transcript being induced by NaCl/KCl treatments in both roots and shoots (Fukuda et al., 2004). *OsNHX1* suppresses Na^+^, Li^+^ and hygromycin sensitivity of yeast *nhx1* mutants and sensitivity to a high K^+^ concentration while overexpression of OsNHX1 in rice imparts salinity tolerance at seedling and reproductive stages (Fukuda et al., 2004; Islam and Seraj, 2009; Biswas et al., 2015; Amin et al., 2016).

Alternative splicing (AS) is an important post-transcriptional gene regulatory mechanism contributing to transcript diversity from the same locus, resulting in the production of two or more transcript isoforms from a given pre-mRNA (Filichkin et al., 2015). Five basic modes of AS are recognized: exon skipping, mutually exclusive exons, intron-retention (IR), alternative donor sites, and alternative acceptor sites (Zhang et al., 2015). Of these, IR events are the most common form of AS observed in plants (Ner-Gaon et al., 2004; Mei et al., 2017). IR in the 5′ UTR or 3′ UTR can affect transcript stability and/or translatability. However, IR between coding exons can lead to loss of ORF integrity, introduction of premature termination codons (PTCs) that can mark transcripts for nonsense mediated decay (NMD; Nicholson et al., 2010). The biological rationale for increased IR during AS of transcripts within coding regions of transcripts in plants is now being increasingly understood. Stimulus-specific reversible sequestration of IR transcripts with subsequent splicing may be a mechanism to prevent targeting by the NMD pathway and rapid protein synthesis in the absence of transcription (Boothby et al., 2013; Gohring et al., 2014; Brown et al., 2015; Filichkin et al., 2015). In *O. sativa*, three splice variants have been reported for *OsNHX1.* Two AS transcripts are generated by splicing out/retention of an intron in the *OsNHX1* 5′ UTR while the third transcript retains the terminal 13th intron, leading to a truncated ORF (Jung et al., 2009).

The genus *Oryza* spans ∼15 million years of evolution, has 27 species and consists of 11 genome types, six of which are diploid (AA, BB, CC, EE, FF, GG) and four that are polyploid (BBCC, CCDD, HHJJ, KKLL; Stein et al., 2018). With a view to understanding conservation of the mentioned AS events in *NHX1* orthologs in the genus *Oryza*, we first examined gene structure, conserved motifs/amino acid residues and promoter motifs in 11 *Oryza* species. Using RT-PCR and RACE analysis we demonstrate that the spliceosomal machinery, governing retention of the 13th intron, appears to have a more ancient origin while that controlling IR involving the 5′ UTR is of more recent origin, and is present only in *Oryza* AA genomes. The latter can be attributed to substantial differences in the sequence of 5′ UTRs of *NHX1* in species belonging to *Oryza* AA genomes vis-à-vis the *Oryza* BB, FF and KKLL genomes. Preferential tissue specific up- or downregulation of the differentially spliced intron retaining/excluding *NHX1* transcripts under salinity is observed in *Oryza* spp.

## Materials and Methods

### Bioinformatic Analysis

*Oryza* spp. *NHX1* sequences were retrieved from Ensembl Plants^1^ using *O. coarctata NHX1* genomic sequence as query (Accession No. JQ796375.1; Kizhakkedath et al., 2015) and searched against *Oryza sativa japonica* (IRGSP-1.0; Supplementary Table S1). The Na^+^/H^+^ exchanger domain was identified in corresponding NHX1 protein sequences using HMMER v 2.28.0^2^, and domain boundaries were manually curated based on multiple sequence alignment (MSA) generated using CLUSTALW2^3^. Phylogenetic relationships were inferred using neighbor joining with *Setaria italica* NHX1 (XP_004958612.1) as the outgroup. Bootstrap values were derived from 1,000 replicate runs and the phylogenetic tree was constructed using MEGA 7 (Kumar et al., 2016).

5′ upstream sequences of *Oryza NHX1s* were retrieved by querying the NCBI whole genome shotgun (WGS) database (*Oryza* subset) using the *O. coarctata NHX1* 5′ upstream region (JQ796375.1). Transcription factor binding sites (TFBS) were predicted using the algorithm Stress-Responsive Transcription Factor DataBase2 (STIF DB2; Naika et al., 2013), with a z-score threshold cut off of ≥ 1.5. A database of Hidden Markov Models of known stress involved TFBS was employed in the STIF algorithm to predict binding sites in 5′ upstream region of genes. TFBS were graphically represented using GSDS 2.0 (Hu et al., 2015).

### Plant Treatments

#### Isolation of Splice Variants From *O. coarctata* and *O. sativa*

*O. coarctata* used in the present study was collected from Pichavaram, Tamil Nadu, India and maintained in a greenhouse (temperature: 25 ± 2°C; 12 h light/12 h dark photoperiod). Two-month-old tillers were transferred to half strength MS medium (Murashige and Skoog, 1962). Following acclimatization, 1-month-old tillers were transferred to MS medium containing 150 mM NaCl. Seeds of *O. sativa* ssp. *japonica cv.* Dinorado (AC41038), *O. sativa* ssp. *indica cv.*IR20 (AC41066), *O. barthii* (AC100498), *O. nivara* (AC100010), and *O. rufipogon* (AC100028) were obtained from CRRI, Cuttack, India while *O. sativa* ssp. *indica cv.* Pokkali (IC324582) was obtained from NBPGR, Thrissur, India. Following dormancy breaking, seeds were sown in vermiculite and irrigated with Yoshida medium (25 ± 1°C; 16 h light/8 h dark; Yoshida et al., 1976) for 2 weeks. The seedlings were then incrementally stressed with NaCl (0, 25, and 50 mM in Yoshida medium) every second day. In both cases, leaf tissues were collected and used for RNA isolation.

#### Plant Treatments for qRT-PCR Analysis

For expression studies, *O. coarctata* (3-month-old) and *Oryza* spp. (25 day-old seedlings) were subjected to incremental salinity stress in 0, 25, 50 and 75 mM NaCl (in Yoshida medium at 3-day intervals) and finally in 100 mM NaCl (4 days). Leaf and root tissues were frozen in liquid nitrogen and used for RNA isolation.

#### Isolation of *rOcNHX1* From *O. coarctata* Genomic DNA

Overlapping *rOcNHX1* genomic fragments were assembled using a combination of (i) genomic PCR (*OcNHX1* cDNA-specific primers; single-step PCR as well as nested PCRs; Supplementary Table S2 and Supplementary Figure S1) and (ii) inverse PCR strategies. For the former, *O. coarctata* genomic DNA (50 ng) was used as template for PCR amplification with *OcNHX1* specific primers (Supplementary Table S2). Amplified *rOcNHX1* fragments corresponding to the *OcNHX1* cDNA size were gel eluted. The amplified product was subjected to electrophoresis in agarose gel from which it was then extracted and purified. Purified fragments were cloned in a T/A vector (*pTZ57R/T*; Fermentas, United States) and sequenced with universal M13 F/R primers. For nested genomic PCRs, the primary PCR reaction was performed in a 20 μL volume with *Pfu* polymerase (Fermentas, United States; 1.25 U). The primary PCR product was diluted (1:40) and 2 μL of template was used for secondary PCR. The 1:20 diluted secondary PCR sample was used for tertiary PCR. PCR products were cloned and sequenced as described before.

For inverse PCR, 100 ng of *O. coarctata* genomic DNA (*Xho*I digestion) was ligated overnight at 16°C with T4 DNA ligase (4U; Fermentas, United States) and precipitated using ethanol. Ligated DNA was used for primary PCR [20 μL reaction volume; 5 μL ligated genomic DNA, 250 nM each dNTP, 200 nM of each oligonucleotide primer; 1.25 U *Pfu* polymerase (Fermentas, United States)]. To increase specificity, secondary and tertiary PCRs were carried out with *OcNHX1*-specific (cDNA junction primers) nested primers. The amplified product was purified, cloned in a T/A vector and sequenced as described above.

#### RNA Isolation and RT-PCR

For samples mentioned in Plant Treatments for qRT-PCR (Materials and Methods), total RNA was isolated using RNAiso Plus (Takara) as per the manufacturer’s protocol. For isolation of the *OcNHX1i13* splice variant (*O. coarctata* 13th intron retaining splice variant), cDNA was synthesized from 1 μg of *DNase1* treated *O*. *coarctata* leaf total RNA. Primer pairs used to amplify the 13th intron retention splice variants from *O*. *coarctata* cDNA are listed in Supplementary Table S2. A “minus RT” (^–^RT-PCR) reaction was included to eliminate genomic DNA contamination in all cases. The fragments were cloned in T/A vector and sequenced. *Oryza* spp. 13th intron retention RT-PCR products were also amplified from *O. sativa* ssp. *japonica cv.* Dinorado, *O. sativa* ssp. *indica cv.* Pokkali, *O. barthii*, *O. glaberrima*, *O. rufipogon*, *O. nivara*, cloned in T/A vector and the fragments from *O. sativa* ssp. *japonica cv.* Dinorado and *O. barthii* sequenced. 5′ UTR splice variants from *Oryza* spp. were amplified from their respective cDNAs using *OsNHX1* Fwd1/Rev1 primers (Supplementary Table S2). Specific fragments (*O. sativa* ssp. *indica cv.* IR20, *O. sativa* ssp. *japonica cv.* Dinorado, *O. barthii*, *O. nivara*) were eluted, cloned in T/A vector and sequenced.

#### 3′ RACE Amplification of *OcNHX1i13*

3′ RACE was performed with the SMARTer RACE 5′/3′ cDNA amplification kit (BD Biosciences, Takara). Template RNA was extracted as described above. 3′ RACE was performed according to the manufacturer’s instructions using nested primers listed in Supplementary Table S2. The primers used for nested PCR were designed against the exon-intron junction [GSP1 (13th exon/13th intron)] and in the 13th intron (GSP2) respectively]. The RACE-PCR product was gel eluted and sequenced.

#### Real-Time Quantitative PCR Analysis (*O. coarctata*; *Oryza* spp.)

First strand cDNA synthesis was performed in a 20 μL reaction volume with 1 μg of total RNA and Superscript III (Invitrogen, United States) at 42°C for 60 min, followed by heat inactivation at 70^*o*^C for 10 min. The primer pairs listed in Supplementary Table S2 were used to amplify fragments of indicated sizes for each splice variant (*Oc-*β*-Actin*; housekeeping gene; internal control). Real time PCR (Quant Studio 6; Thermo Fisher Scientific, United States) was carried out in a reaction volume of 10 μL [1 μL of cDNA, 5 μL of Takara TB Green^TM^ Primix Ex Taq^TM^ II (2X), 0.5 μL each of a given primer pair (final concentration 250 nM each) under the following cycling conditions: 95°C (30 s), 40 cycles of denaturation at 95°C (5 s), annealing and extension at 60°C (30 s) in a 96-well optical reaction plate (Thermo Fisher Scientific, United States). Each real time PCR reaction was performed in triplicate, in order to evaluate data reproducibility for three biological replicates. Amplicon specificity was verified by melt curve analysis (60–95°C at 40 cycles) and subsequent agarose gel electrophoresis. Gene expression was quantified using the comparative CT (2^–Δ^ ^Δ^ ^CT^) quantitation method for all splice variants (*O. coarctata* and *Oryza* spp.) in leaf and root tissues, with values representing “n”-fold difference relative to the housekeeping control gene.

## Results

### The *NHX1* Gene Shows Conservation of Exon Number and Key Amino Acid Residues Within the Encoded NHX Na^+^/H^+^ Exchanger Domain Across *Oryza* Species

There is substantial variation in the lengths of the genomic and coding regions of the eleven *Oryza* spp. *NHX1* sequences examined (Supplementary Figure S2 and Supplementary Table S1). A maximum genomic length of 4,535 bp was observed for *O. brachyantha*, followed by 4,472 bp for *O. longistaminata* and the shortest sequence length of 4,103 bp was observed for *O. coarctata*. Of all the *Oryza* NHX1 amino acid sequences examined, a maximum of length 540 amino acid residues was observed for *O. coarctata* and the shortest of 505 amino acids for *O. longistaminata* (Supplementary Table S1). All *Oryza NHX1* genes examined showed a conserved exon-intron organization: *viz.*14 exons and 13 introns (Supplementary Figure S2), with the second and the thirteenth (last) introns being the longest.

**FIGURE 1:**
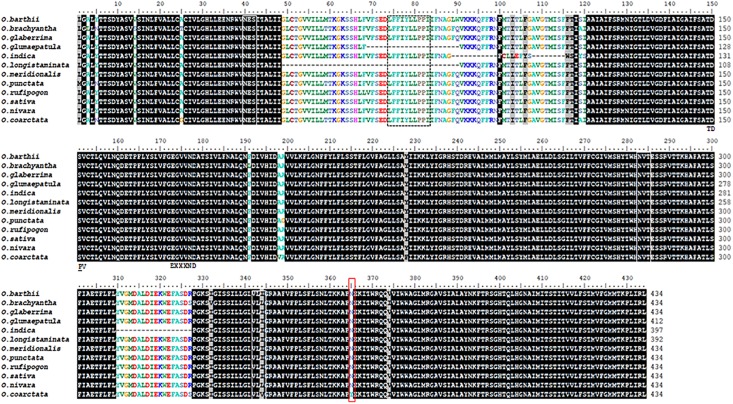
Alignment of the Na^+^/H^+^ exchanger domain found in NHX1 protein sequences (*Oryza* species). The Na^+^/H^+^ exchanger domain length is approximately 443-446 amino acid residues (*O. longistaminata* and *O. glumaepatula* are exceptions). The Na^+^/H^+^ inhibitory amiloride binding domain “LFFIYLLPPI” is boxed (dotted line). Putative glycosylation sites are conserved and indicated by white boxes (“NES,” “NVT,” and “VLI/V/L”). Motifs implicated in antiporter function: “A_1_T_2_D_3_S_4_” (marked TDPV), “E_5_*---*N_6_D_7_” (marked EXXND), serine and arginine (R_8_) residues (marked *) are also absolutely conserved across *Oryza* species. These motifs and residues occur on different transmembrane helices that form part of the protein core containing the substrate binding site (Masrati et al., 2018). The amino acid residues boxed in red are conserved across *Oryza* species except in *O. coarctata*.

A single Na^+^/H^+^ exchanger domain belonging to the sodium/hydrogen exchanger family is identifiable in all the *Oryza* species examined using HMMER (Supplementary Figure S3). Domain boundaries were manually curated by multiple alignment; nine of the *Oryza* spp. NHX1 have similar domain lengths (434 amino acid residues), with *O. longistaminata*, and *O. glumaepatula* showing shorter domain lengths of 392 and 412 amino acid residues, respectively (Figure 1). Both *O. longistaminata* and *O. glumaepatula* sequences show deletions (42 and 22 amino acid residues, respectively) in the region centered around and inclusive of the Na^+^/H^+^ inhibitory amiloride binding domain “LFFIYLLPPI.” Two of three identified putative glycosylation sites are absolutely conserved (“NES”; “NVT”) across all *Oryza* species while the third site shows variation at the third amino acid residue (VLI/V/L). In addition, motifs “A_1_T_2_D_3_S_4_,” “E_5_*---*N_6_D_7_,” serine and arginine (R_8_) residues (marked ^∗^) are also absolutely conserved across the *Oryza* species. These motifs and residues do not occur sequentially but appear on different transmembrane helices that form part of the protein core containing the substrate binding site and have been implicated in antiporter function (with the exception of serine marked with an asterisk; Masrati et al., 2018).

Phylogenetic analysis of the NHX1 domain protein sequences recognizes two main clades in which all the *Oryza* spp. NHX1 sequences belonging to AA genomes (expect *O. nivara*) cluster together while *O. coarctata* (KKLL), *O. brachyantha* (FF), and *O. punctata* (BB) NHX1 sequences cluster separately and are more closely related (Supplementary Figure S4).

### Analysis of the 5′ Upstream Sequences of *Oryza NHX1* Identifies a Conserved Order of *cis-*Elements That Are Implicated in Abiotic Stress Responsiveness

**FIGURE 2:**
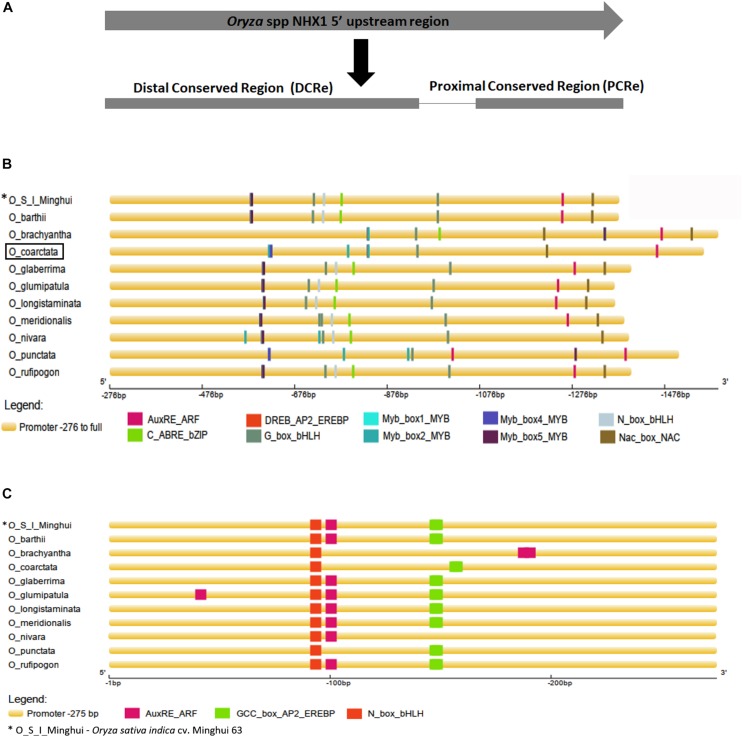
Analysis of 5′ upstream sequences from *Oryza* species. **(A)** Alignment of NHX1 5′ upstream sequences from *Oryza* species shows two conserved stretches (i) Region 1, starting -276 bp upstream of the NHX1 5′ UTR to the end of examined sequence (*O. coarctata* NHX1 promoter sequence used as reference for numbering) and (ii) Region 2 encompassing bases -1 to -275 bp, upstream of the NHX1 5′ UTR. **(B,C)** Prediction of transcription factor binding *cis*-elements using STIF DB2 (Naika et al., 2013) in the 5′ upstream sequences of NHX1 (*Oryza* species), Region 1 and Region 2 respectively, aligned NHX1.

Aligned 5′ upstream sequences of *Oryza* spp. *NHX1* (Supplementary Figure S5) showed variation in length, with a maximum size of 1,590 bp (*O. brachyantha*) and a minimum size of 1,367 bp (*O. barthii*). In eight of the eleven sequences examined, two conserved regions were identified: (i) the proximal conserved region (PCRe) encompassing bases -1 to -275 bp upstream of *NHX1* “ATG” start codon (*O. coarctata* promoter sequence used as reference for numbering; inclusive of 5′ UTR) and (ii) the distal conserved region (DCRe), starting -276 bp upstream of the *NHX1* gene to end of the analyzed sequence (upstream) examined (Supplementary Figure S5 and Figure 2A). Transcription factor (TF) binding *cis*-motifs were predicted using the STIF algorithm (Naika et al., 2013) and 16 *cis*-elements each were identified in *O. glumaepatula* and *O. meridionalis* while 12 *cis*-elements each were identified in *O. coarctata* and *O. punctata*.

Multiple *cis-*elements were clustered within the DCRe of the *Oryza NHX1* gene 5′ upstream sequences analyzed (Figure 2B). Based on the *cis-*elements and their relative positions/order within the DCRe, sequences from *Oryza* AA genomes cluster into one group while *O. coarctata, O. brachyantha* and *O. punctata* share a similar organization of *cis-*elements in their 5′ upstream sequences. In the *Oryza* AA genome, *NHX1* 5′ upstream sequences, *cis-*elements were found in the order (-275 to end): (i) overlapping Myb/DREB binding element; (ii) Gbox-HLH; (iii) Nbox-HLH binding site; (iv) ABRE/bZIP; (v) Gbox-HLH binding site; (vi) AuxRE; and (viii) NAC binding site. *O. coarctata*, *O. brachyantha* and *O. punctata NHX1* 5′ upstream sequences (-275 to end) show: (i) one NAC binding site; (ii) one AuxRE; (iii) one Gbox-HLH; and (iv) numerous Myb TF binding sites. All eleven sequences show the presence of a highly conserved distal Gbox-HLH motif, ten have *NHX1* 5- upstream sequences and, in addition, show conservation of the AuXRE (*O. nivara* is an exception) and nine sequences show conservation of a distal NAC TF binding region in the DCRe.

Within the PCRe, seven *Oryza NHX1* gene upstream sequences (Figure 2C) show the presence of a conserved N_box_bHLH *cis*-element, the AuxRE and the GCC_box_AP2_EREBP *cis*-element. With the exception of *O. nivara*, sequences from *Oryza* AA genomes cluster together, based on the *cis*-elements and their relative positions/order within the PCRe.

### Alternative Splicing of an Intron in the 5′ UTR of the *NHX1* Gene in *Oryza* AA Genomes Leads to Two Distinct Transcripts

**FIGURE 3:**
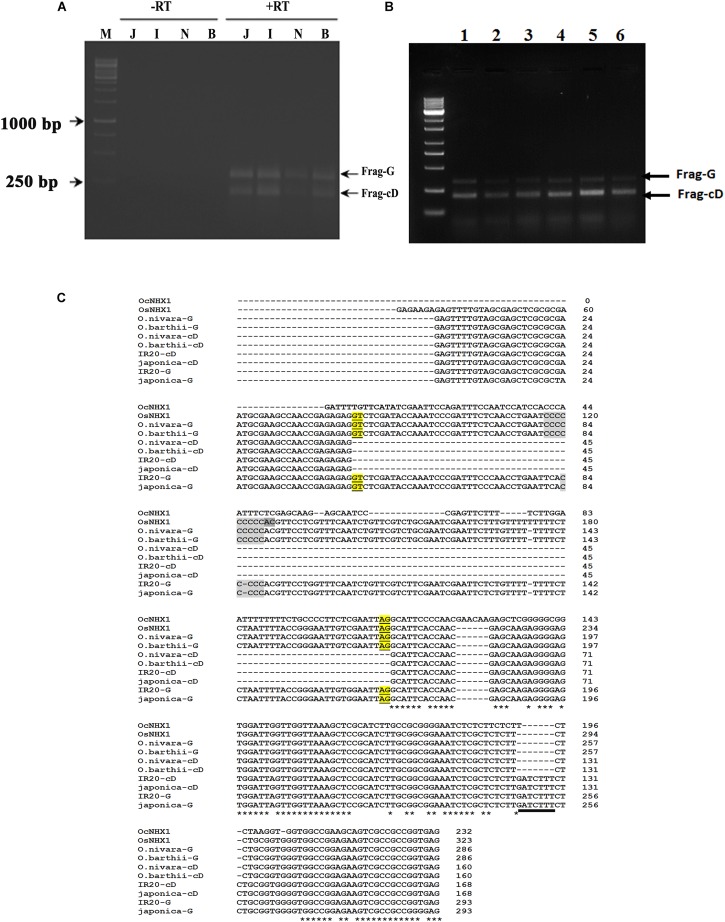
Cloning of 5′ UTR NHX1 splice variants from *Oryza* AA genome types. **(A)** RT-PCR analysis of NHX1 5′ UTR splice variants from *O. sativa* [ssp. *japonica* (J), cv. IR20 (I), *O. nivara* (N), and *O. barthii* (B) that either retain the first intron (Frag-G) or splice it out (Frag-cD). M- Marker, Letter on top of each image indicate the rice spices; J-*Japonica*, I-*indica* IR20, N-*O. nivara*, and B-*O. barthii*; -RT: PCR using DNase treated RNA, + RT: PCR using cDNA. **(B)** RT-PCR analysis of NHX1 5′ UTR splice variants from *O. barthii* (1), *O. nivara* (2), *O. glaberrima* (3), *O. sativa* [ssp. *japonica* (4)], *O. sativa* [ssp. *indica* cv. *Pokkali* (5)], *O. rufipogon* (6); (Frag-G), and (Frag-cD) are indicated. **(C)** RT-PCR fragments shown in **(A)** were cloned and sequenced. The *O. sativa* NHX1 cDNA fragments (cv. *japonica* and *IR20*) group together and show two distinct differences from *O. nivara* and *O. barthii* cDNA fragments: (i) length of a “C” repeat region in the intron and (ii) the presence of a “GATCTTT” insertion in the second exon that is underlined (5′ UTR); the “GATCTTT” insertion also occurs in *O. rufipogon* and *O. longistaminata* (Supplementary Figure S6).

The 5′ UTR of *NHX1* is highly conserved in *Oryza* species containing the AA genome (Supplementary Figure S6) and includes a conserved intron that is either retained or spliced out to give two distinct *O. sativa OsNHX1* transcripts (Jung et al., 2009). We examined if the alternative splicing events observed for the 5′ UTR of *NHX1* are conserved in other *Oryza* species containing AA genomes. RT-PCR analysis of *O. barthii* and *O. nivara*, *O. rufipogon*, *O. sativa* (cvv. IR20 and Pokkali) using leaf tissues and *NHX1* 5′ UTR-specific primers (*OsNHX1* Fwd/*OsNHX1* Rev1) amplified one fragment showing IR (labeled Frag-G) and a second transcript, termed Frag-cD, in which the first intron was spliced out (Figure 3A; marked + RT). *DNase* I treatment was used to eliminate genomic DNA contamination prior to RT-PCR (Figure 3A; marked -RT). The two cDNA fragments (Frag-G/Frag-cD) obtained for *O. sativa* (cv. *japonica* and cv. *IR20*), *O. nivara*, and *O. barthii* are shown in Figure 3B. RT-PCR products corresponding to Frag-G and Frag-cD were cloned for *O. sativa* cv. IR20 and *O. barthii* (Figure 3C). BLAST analysis confirmed in all cases that the cloned fragments corresponded with *OsNHX1*. The 5′ UTR sequence of *NHX1* examined in the *Oryza* AA species shows a high degree of conservation including the intron. The *O. sativa* cDNA fragments (cv. *japonica* and *IR20*) group together and show two distinct differences from *O. nivara* and *O. barthii*: (i) length of a “C” repeat region in the intron differs and (ii) the presence of a “GATCTTT” insertion in the second exon (also part of the 5′ UTR; underlined).

### The *OcNHX1i13* Transcript From Wild Rice *O. coarctata* Shows Retention of the 13th Intron

**FIGURE 4:**
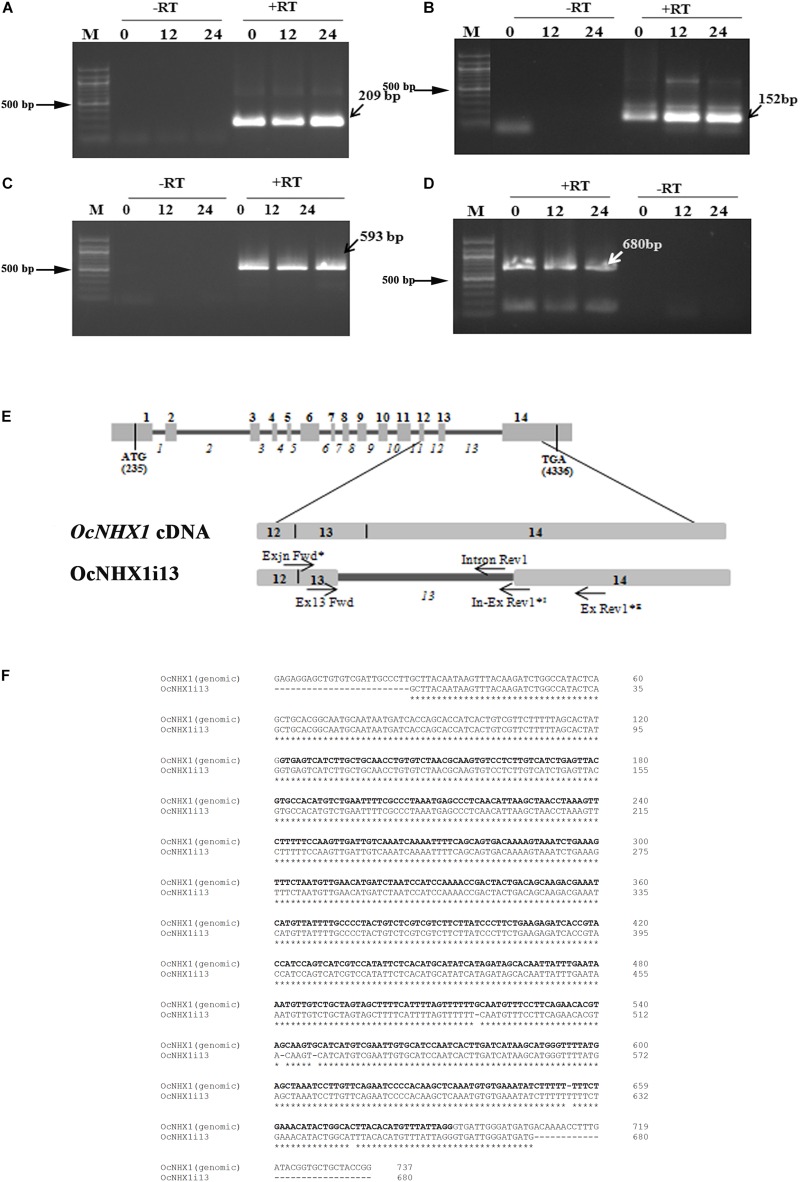
Cloning of the 13th intron retaining splice variant from *O. coarctata.* Complete retention of the 13th intron in *O. coarctata* is confirmed by RT-PCR amplification using primer pairs listed in Supplementary Table S2, generating fragments of sizes **(A)** 209 bp; **(B)** 152 bp; **(C)** 593 bp, and **(D)** 680 bp (*OcNHX1il3*), respectively. In all cases a “RT-PCR” reaction was included to rule out genomic DNA contamination. **(E)** Schematic representation showing alignment of the *OcNHX1il3* RT-PCR product with the corresponding *OcNHX1* genomic region. Primer pairs used to isolate *OcNHX1il3* by RT-PCR are indicated by an asterisk (*). **(F)** Alignment of *OcNHX1il3* and corresponding *OcNHX1* genomic sequence. The 680 bp fragment showed near 100% identity with the corresponding O*cNHX1* genomic sequence (Accession no: JQ796375.1). The 13th intron is highlighted in bold.

Of the three *OsNHX1* transcripts reported, *Os07g47100.3* (*OsNHX1*; MSU annotation; *japonica*) shows retention of the 13th intron. RT-PCR analysis of salinity-treated *O. coarctata* leaf tissues (150 mM NaCl) confirmed that the IR splicing event observed for *OsNHX1* (13th intron) is also conserved in *O. coarctata* (*OcNHX1i13*; 680 bp); this was further confirmed by RT-PCR amplification of fragments of varying sizes using primers flanking the 13th intron (209, 680, 593, and 152 bp; Figures 4A–E). The 680 bp fragment showed 100% identity with the O*cNHX1* genomic sequence: Accession no: JQ796375.1 (Kizhakkedath et al., 2015; Figure 4F). The 3′ RACE PCR *OcNHX1i13* product shows complete retention of the 13th intron and 14th exon (Supplementary Figure S7).

### Retention of the 13th Intron in *NHX1* Transcripts Is Also Observed in *Oryza* Species AA Genomes

**FIGURE 5:**
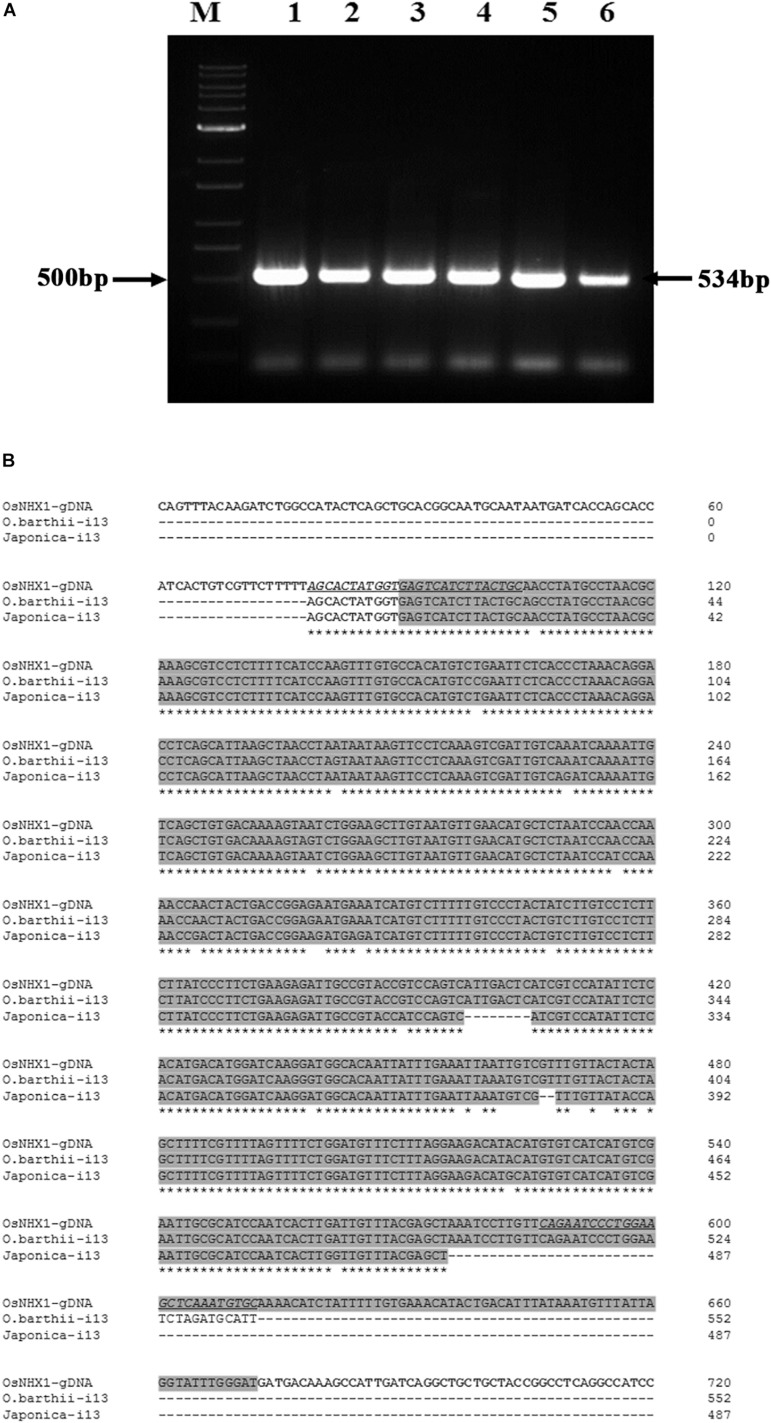
Cloning of NHX1 13th intron retaining splice variants from *Oryza* AA genome types. **(A)** RT-PCR (DNAse I treated RNA) amplification of 13th intron retaining NHX1 fragments from *O. barthii* (1), *O. nivara* (2), *O. glaberrima* (3), *O. sativa* [ssp. *japonica* (4)], *O. sativa* [ssp. *indica* cv. *Pokkali* (5)], *O. rufipogon* (6); Expected fragment size of 534 bp *O. sativa* [ssp. *japonica*] is indicated. Primers were designed to target the 13th intron of NHX1 in all cases (12th exon-13th intron junction forward and 13th intron reverse primer. **(B)** Sequence alignment *OsNHX1* genomic DNA and RT-PCR fragments from *O. sativa* [ssp. *japonica*] and *O. barthii* from **(A)** were sequenced to show retention of the 13th intron. Primer sequence is underlined and the intron region is shaded gray.

RT-PCR products from the *Oryza* species containing the AA genome, *O. barthii*, *O. glaberrima* and *O. nivara NHX1* (amplified by primers flanking the 13th intron), also show retention of the 13th intron (based on a larger amplified fragment size obtained; expected size based on *OsNHX1*: 534 bases). In addition, retention of the 13th intron was also confirmed in *O. sativa* subspp. *japonica* and *O. sativa* subspp. *indica* cv. Pokkali by RT-PCR (Figure 5A). Subsequent cloning and sequencing of *O. barthii* and *Oryza sativa* subspp. *japonica* RT-PCR products confirmed complete retention of the 13th intron (Figure 5B).

### The *O. coarctata* Genome Shows the Presence of a Retrocopy (*rOcNHX1*) of the *OcNHX1* cDNA

**FIGURE 6:**
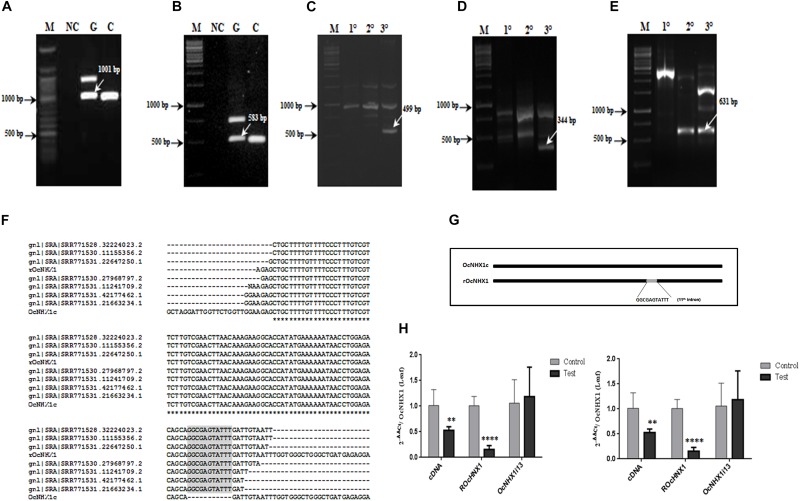
Isolation of *rOcNHX1* from *O. coarctata*. **(A–E)** A 2,032 bp size *OcNHX1* retrogene (*rOcNHX1*) was isolated from *O. coarctata* genomic DNA using a combination of genome walking techniques (also Supplementary Figure S1). **(A,B)** PCR with *OcNHX1* specific primers and exon-exon junction primers used for gene isolation (1001 and 583 bp) **(C)** 499 bp fragment was isolated by Inverse PCR (*Xho*I enzyme) using nested primers. **(D,E)** 344and 602 bp amplicons were isolated by nested PCR. M- DNA ladder; NC-Negative control; G- *O. coarctata* genomic DNA; C- *O. coarctata* cDNA; 1^0^ – Primary PCR; 2^0^ –Secondary PCR; 3^0^ – Tertiary PCR. **(F)** *rOcNHX1* differs from the *OcNHX1* cDNA only in having (i) 11-bp insertion corresponding to the part of the 5′ splice site of the 11th intron in the *OcNHX1* genomic sequence (ii) 2 single nucleotide polymorphisms. **(G)** Analysis of the sequence read archive (SRA; *O. coarctata* transcriptome; Garg et al., 2013) confirmed the *rOcNHX1* expression across stress treatments (450 and 700 mM NaCl, Submergence, Submergence + 450 mM NaCl). **(H)** qRT-PCR expression analysis of *OcNHX1* splice variants in *O. coarctata* leaf and root tissues respectively under salinity. cDNA-correctly spliced *OcNHX1* cDNA; *rOcNHX1*: *OcNHX1* retrocopy; *OcNHX1i13- OcNHX1* splice variant with retention of 13th intron. Data presented is the mean of three biological replicates (*n* = 3; with each replicate analyzed twice). Significance was calculated using one-way ANOVA (Student’s *T*-test). Statistical significance (*p*-value) : *P < 0.05; **P < 0.01, ***P < 0.001, ****P < 0.0001.

While performing a routine PCR with *O.* c*oarctata* genomic DNA, multiple fragments were amplified using primer pair Seq Fwd and Seq Rev (Supplementary Table S2). Of these, one corresponded exactly in size with *OcNHX1* cDNA. This fragment was cloned and sequenced and was found to be identical to the *OcNHX1* cDNA except for a 11 bp sequence insertion, corresponding to the 5′ end of the 11th intron of the *OcNHX1* genomic sequence. Subsequent PCR amplifications using combinations of exon-exon junction primers suggested that a copy of the *OcNHX1* cDNA may be inserted in the *O. coarctata* genome (Supplementary Figure S1). Using a combination of genome walking techniques, a 2,032 bp sequence corresponding to the *OcNHX1* retrogene (*rOcNHX1*) was isolated from *O. coarctata* genomic DNA (Figures 6A–E and Supplementary Figure S8). *rOcNHX1* differs from the *OcNHX1* cDNA in having an 11 bp “GGCGAGTATTT” insertion identical to a region flanking the 5′ splice site of the 11th intron in the *OcNHX1* genomic sequence (Figure 6G) and two single base pair changes. Analysis of the sequence read archive (SRA; *O. coarctata* transcriptome; Garg et al., 2013) confirmed the expression of *rOcNHX1* in salinity stress treatments (450 and 700 mM NaCl, Submergence, Submergence + 450 mM NaCl; Figure 6F).

### Alternatively Spliced *O. coarctata* and *Oryza* spp. *NHX1* Transcripts Show Tissue Specific Switches in Isoform Expression Under Salinity

In *O. coarctata* leaf tissues, expression of the correctly spliced *OcNHX1* cDNA and its retrocopy, *rOcNHX1* is significantly reduced (*P* < 0.001 and *P* < 0.0001) under salinity stress while the 13th intron retaining transcript, *OcNHX1i13*, shows increased expression (Figure 6H). In contrast, in root tissues under salinity stress, expression of the correctly spliced *OcNHX1* cDNA and *rOcNHX1* (*P* < 0.0001) is upregulated and the 13th intron retaining transcript, *OcNHX1i13* is downregulated (*P* < 0.001; Figure 6H).

**FIGURE 7:**
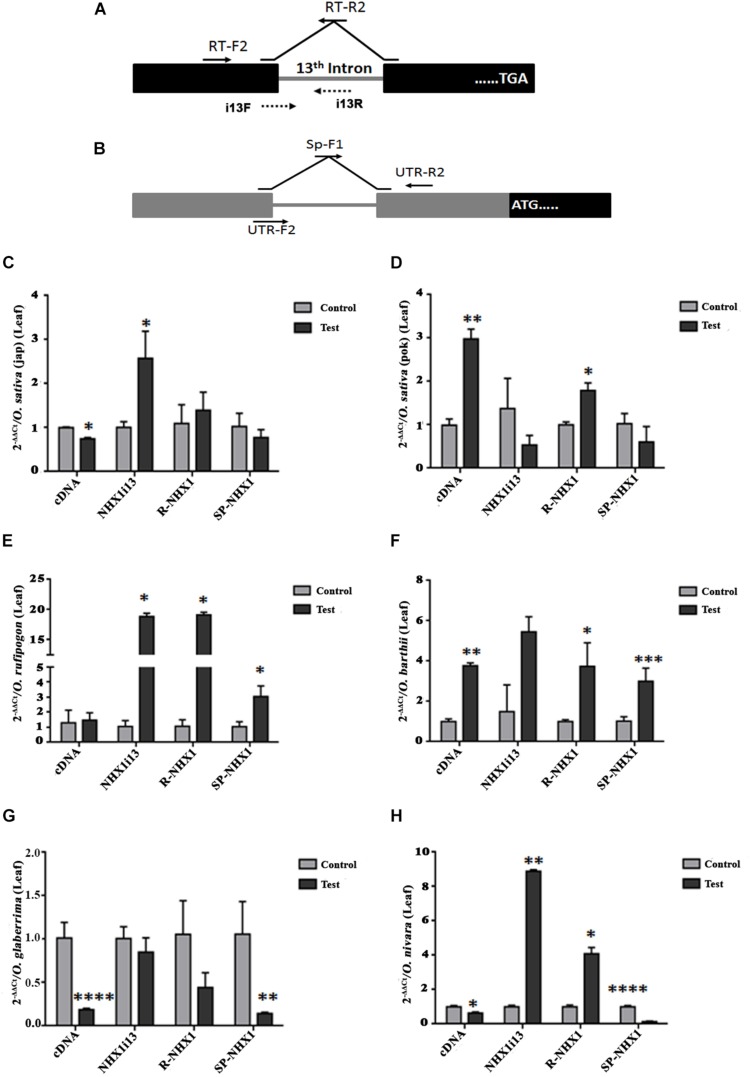
qRT-PCR expression analysis of *NHX1* splice variants in *Oryza* spp. leaf tissues under salinity. The following splice variants were examined: correctly spliced transcript (cDNA), 13th intron retaining transcript (NHXi13), the 5′ UTR intron retaining NHX1 transcript (R-NHX1) and the 5′ UTR intron splicing NHX1 transcript (Sp-NHX1). **(A)** For the correctly spliced *NHX1* transcript 12th exon forward primer (RT-F2) and 13th–12th exon reverse (RT-R2) primers were used (Kizhakkedath et al., 2015). For the 13th intron retention transcript i13F (12th exon-13th intron junction) and i13R (13th intron) primers were used. **(B)** For the 5′ UTR intron retaining/splicing transcripts primer pairs (UTR-F2 and UTR-R2; Sp-F1 and UTR-R2 respectively) were use (Supplementary Table S2). **(C–H)** *Oryza* spp. examined: *O. sativa* [ssp. *japonica* **(C)**], *O. sativa* [ssp. *indica* cv. Pokkali **(D)**], *O. rufipogon* **(E)**, *O. barthii* **(F)**, *O. glaberrima* **(G)**, *O. nivara* **(H)**. All qRT-PCRs for a given sample (splice variants) were analyzed on the same plate under identical reaction conditions from the same cDNA batch. Data presented is the mean of three biological replicates (*n* = 3; with each replicate analyzed twice; each replicate had 5 pooled plants). Significance was calculated using one-way ANOVA (Student’s *T*-test). *P < 0.05; **P < 0.01, ***P < 0.001, ****P < 0.0001.

**FIGURE 8:**
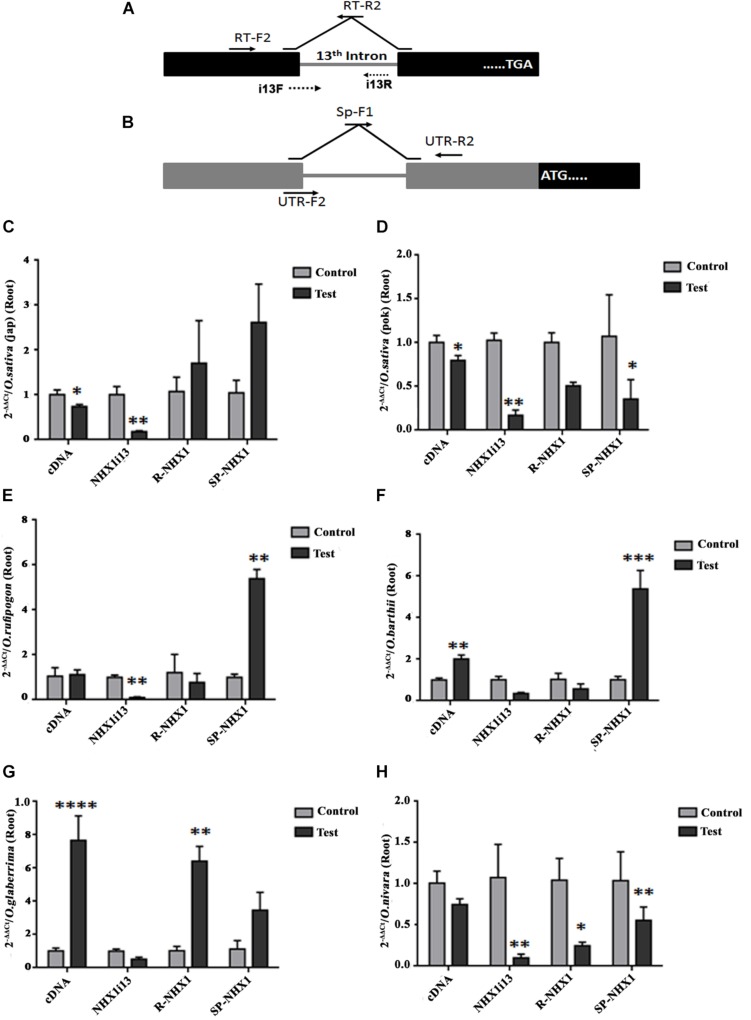
qRT-PCR expression analysis of *NHX1* splice variants in *Oryza* spp. root tissues under salinity. **(A,B)** Same as in Figures 7A,B. **(C–H)** *Oryza* spp. examined: *O. sativa* [ssp. *japonica* **(C)**], *O. sativa* [ssp. *indica* cv. Pokkali **(D)**], *O. rufipogon* **(E)**, *O. barthii* **(F)**, *O. glaberrima* **(G)**, *O. nivara* **(H)**. All qRT-PCRs for a given sample (splice variants) were analyzed on the same plate under identical reaction conditions from the same cDNA batch. Data presented is the mean of three biological replicates (*n* = 3; with each replicate analyzed twice; each replicate had 5 pooled plants). Significance was calculated using one-way ANOVA (Student’s *T*-test). *P < 0.05; **P < 0.01, ***P < 0.001, ****P < 0.0001.

We also examined expression of the correctly spliced NHX1 transcript, the 13th intron retaining transcript (NHXi13), the 5′ UTR intron retaining NHX1 transcript (R-NHX1) and the 5′ UTR intron splicing NHX1 transcript (Sp-NHX1) in leaf and root tissues (Figures 7, 8 and Supplementary Figures S9, S10) of four *Oryza* AA genome-type species (*Oryza sativa* subspp. *japonica*, *Oryza sativa* subspp. *indica cv.* Pokkali, *O. rufipogon*, *O. barthii, O. glaberrima*, and *O. nivara*) under salinity, the salient features for which are summarized as:

(1) *O. sativa* varieties show variability in tissue specific expression of differentially spliced *NHX1* isoforms under salinity (*japonica*: downregulation and Pokkali: upregulation in leaf of the correctly spliced isoform; downregulation in roots of both varieties under salinity).
(2) The 13th intron retaining NHX1 isoform (i13) is upregulated in *Oryza* spp. leaf tissues (except *Oryza sativa* spp. *indica cv.* Pokkali and *O. glaberrima*) and downregulated in root tissues of *Oryza* spp.
(3) The 5′ UTR intron retaining NHX1 (R-NHX1) is upregulated in leaf tissues of *Oryza* spp. (except for *O. glaberrima*) and downregulated in root tissues (except in *Oryza sativa* spp. *japonica* and *O. glaberrima*).
(4) The 5′ UTR intron splicing NHX1 transcript (Sp-NHX1) shows downregulation in *Oryza* spp. under salinity in leaf tissues (exceptions are *O. rufipogon* and *O. barthii* and upregulation in root tissues (exceptions include *Oryza sativa* spp. *indica cv.* Pokkali, and *O. nivara*).

## Discussion

Plant NHXs occur as multigene families, and recent data from *Arabidopsis* triple and quadruple vacuolar NHX knockouts suggests they have a central role in cellular cation homeostasis that impacts growth and development (Bassil et al., 2019). Cultivated rice, *O. sativa*, has a multiplicity of NHX transporters (Fukuda et al., 2011). Of these, *OsNHX1* has been studied in more detail (Fukuda et al., 2004; Islam and Seraj, 2009; Biswas et al., 2015; Amin et al., 2016).

### *Oryza* NHX1 Orthologs Show Conservation of Critical Amino Acid Residues That Are Vital for Antiporter Function and Characteristics of Type I Cation/Proton Antiporters (CPAs)

Orthologs of NHX1 in *Oryza* species show conservation of the Na^+^/H^+^ exchanger domain, with NHX1 sequences from AA *Oryza* genomes forming a distinct cluster. Eight critical residues governing the function of EcNhaA antiporter in *Escherichia coli*, in the “NhaA fold” (X_1_X_2_X_3_X_4_-*---*E_5_XXN_6_D_7_*------*R_8_), occur clustered in the protein core. They reside on different transmembrane (TM) helices and do not occur necessarily in a continuous manner in the protein sequence (Padan and Michel, 2015; Masrati et al., 2018). These residues are highly conserved in *Oryza* NHX1s. The amino acid residues “ATDS” in *Oryza* NHX1 species form the first four positions that occur continuously (TM4 of *EcNhaA*) and are spaced at a distance of 20 amino acid residues from the crucial “E_5_XXN_6_D_7_” motif (TM5 of *EcNhaA*). In fungal antiporters (*Zygosaccharomyces rouxii* ZrSod2-22p; *Saccharomyces pombe* SpSod2p; *S. cerevisiae* ScNha1p; *Candida albicans* CaCnh1p; *C. tropicalis* CtNha1p; *Pichia sorbithophila* PsNha1p), the motif “ATDP” is found instead of “ATDS” seen in *Oryza* species; replacement of proline by serine/threonine abolishes Na^+^ specificity and confers a broader cation transport ability on ZrSod2-22p (Kinclova-Zimmermannova et al., 2006). Similarly, a single amino acid change (Asp) in the second pore loop domain of *Thellungiella salsuginea* TsHKT1;2, a class 1 HKT protein, confers strong selectivity for K^+^ transport over Na^+^; the corresponding residue in other class 1 HKT transporters is Asn (Ali et al., 2012). Based on presence of the “ATDS” motif, a broader cation substrate specificity for Oryza NHX1 proteins is predicted. Thus, OsNHX1 shows broad substrate preference and suppresses Na^+^, Li^+^, and K^+^ sensitivity of the yeast *nhx1* mutant while *O. coarctata* (OcNHX1) suppresses Na^+^, but enhances Li^+^ sensitivity of a yeast *nhx1* mutant (Fukuda et al., 2004; Jegadeeson et al., 2019). Within the E_5_XXN_6_D_7_ motif, E_5_ is highly conserved in type I CPAs, with N_6_ and D_7_ being vital for ion binding and translocation. The eighth residue, R8 (marked ^∗^) is 167 amino acid residues downstream from the E_5_XXN_6_D_7_ motif. It corresponds to K300 of *EcNhaA* (TM10), and it has been implicated in maintenance of the NhaA fold (Rimon et al., 2018). The crucial N_6_D_7_ residues have been experimentally verified as being important for transporter function in poplar NHX3 (*PeNHX3*) and are also conserved in AtNHX1 (Wang et al., 2014; Sze and Chanroj, 2018).

### 5′ Upstream Regions of *Oryza NHX1* Show a Conserved Order of *cis*-Elements

Multiple, conserved *cis*-elements were predicted using the STIF algorithm in the 5′ upstream region (in DCRE and PCRe regions) of *Oryza NHX1* sequences. A nucleotide sequence characteristic of a Gbox HLH binding site (DCRe) and a Nbox HLH binding motif (proximal end) is conserved in all sequences. In addition, ten *NHX1* 5′ upstream sequences show conservation of a distal AuxRE binding site, while nine sequences show conservation of a distal NAC TF binding region. In the PCRe, an AuxRE and a GCC_box_AP2_EREBP *cis*-motif are also conserved in seven *Oryza* species. HLH, NAC and AP2/EREBP transcription factors have well-documented roles under salinity stress (Zhang and Shi, 2013; Chen et al., 2016), while AuxRE bind Auxin Response Factors (ARFs) which are known to be induced in response to salinity (Qi et al., 2012; Bouzroud et al., 2018). The cladogram derived from the order of predicted *cis*-elements reveals that *Oryza* species with AA genomes have a distinct pattern of cis-elements that are distinct from BB, FF and KKLL genome types. In *O. sativa*, *OsNHX1* expression is induced in response to hyper-ionic stress (NaCl, KCl) stress in both shoots and roots (Fukuda et al., 2004). Our data too suggests responsiveness to NaCl application in *O. sativa* and *O. coarctata*. However, NHXs have broader roles in plant function including cell growth, expansion, cellular trafficking in relation to environmental cues. It is worth examining this clustering of cis elements vis-à-vis tissue specific/stimulus specific gene expression.

### Sequence Divergence of the 5′ UTR in *Oryza* Species Determines Splicing Outcomes

For the first time, in this work, we demonstrate experimentally the existence of 5′ UTR splice variants for *OsNHX1* and conservation of the 5**′** UTR splicing event in the *NHX1* gene in three cultivated *Oryza* progenitor genomes (*O. nivara*, *O. rufipogon*, and *O. barthii* all AA genomes). Diversity in the genus *Oryza* arose as a consequence of two rapid speciation events that occurred approximately 13–15 million years ago (MYA), wherein the present G, F, and extant H/J/K/L *Oryza* genome types separated from an ancestral species that gave rise to the *Oryza* A, B, C genome types (Zou et al., 2013; Stein et al., 2018). The AA genomes are thought to have developed in a second burst of speciation about 2.41 MYA (Stein et al., 2018). Divergence of African cultivated rice, *O. glaberrima* (progenitor species, *O. barthii*) from Asian cultivated rice *O. sativa* (progenitor species, *O. rufipogon* and *O. nivara* for *O. sativa* ssp. *indica* and *O. sativa* ssp. *japonica*, respectively), occurred about 0.86 MYA, from an extinct common *Oryza* ancestor (Zhu et al., 2014). The presence of the 5′ UTR intron containing/splicing out variants in the African rice progenitor, *O. barthii*, and *O. nivara* and *O. rufipogon* (O. sativa progenitor species) suggests that the transcriptional machinery governing these splicing events has a conserved, ancient origin, dating back to at least 0.86 MYA and was probably inherited from a shared common ancestor. Further, alternative splicing events involving the 5′ UTRs of *NHX1* transcripts are conserved in *Oryza* AA species.

The *NHX1* 5′ UTR regions of *Oryza* BB, CC and FF genome types (*O. punctate*, BB genome type, accession No: KC610950.1; *O. officinalis*, CC genome type, accession No: KC610951.1; *O. brachyantha*, FF genome type, accession No.:XM_006658016.2); and *O. coarctata* (KKLL) (accession no: JQ796375.1; Kizhakkedath et al., 2015; Supplementary Figure S11) show substantial sequence differences from the *NHX1* 5′ UTR regions of *Oryza* AA genome sequences. This may account for why we were able to detect only one transcript for *OcNHX1* by RT-PCR (using 5′ UTR specific primers). Based on sequence comparisons, we predict that this splicing event is likely absent in *NHX1* transcripts of *Oryza* BB and FF genome types and possibly other *Oryza* genome types as well.

### Alternative Splicing Involving Retention of the 13th Intron in *Oryza NHX1* Transcripts Is More Ancient in Origin Compared to the 5′ UTR Splicing Events

MSU annotation of the *Os07g47100.3* (*OsNHX1*) transcript suggests partial retention of the 13th intron. However, the *O. coarctata OcNHX1i13* transcript not only shows complete retention of the 13th intron but also the entire last is exon (confirmed by 3′ RACE-PCR; Supplementary Figure S7). Analysis of the Transcriptome Shotgun Assembly (TSA) database at NCBI suggests that transcripts showing complete retention of the 13th intron may also be present in *O. sativa* and *O. meyeriana* (GG genome type) transcriptomes [GFYC01000051.1; GFMS01046554.1 (*O. sativa*); GBYS01092482.1; GBYS01172993.1 (*O. meyeriana*)]. The 3′ splice site in 13th intron of the *OcNHX1* genomic sequence is “GG” instead of the canonical “AG” seen in *OsNHX1* genomic DNA (canonical AG present in the remaining *NHX1* sequences). The 13th intron is the last intron seen in both *OcNHX1* and *OsNHX1* genomic sequences. IR appears to constitute a significant proportion of alternatively spliced transcripts observed in plants and in numerous cases is induced under abiotic stress conditions (Ner-Gaon et al., 2004; Filichkin et al., 2018). Further, in a large majority of transcripts, it is the last intron that is usually retained, similar to *Os07g47100.3* and *OcNHX1i13* (Ner-Gaon et al., 2004). The presence of transcripts retaining the 13th intron in *Oryza* AA, KKLL, and GG genome types suggests that this splicing event may have a more ancient origin that pre-dates the alternative splicing event involving the *NHX1* 5′ UTR in *Oryza* AA species.

### *NHX1* ORF and Transcript Diversity Generated by Alternative Splicing Across *Oryza* Species

Splicing usually occurs co-transcriptionally and preferential or position-specific intron retention during alternative splicing is influenced by DNA methylation in rice (Wei et al., 2018) as well as chromatin remodeling in rice and *Arabidopsis* (Ullah et al., 2018). Our data shows preferential tissue specific up- or downregulation of the correctly spliced *NHX1* transcript as well as splice variants under salinity in *Oryza* AA species as well as in *O. coarctata*. This suggests the spliceosome machinery governing alternative splicing events involving *NHX1* has tissue specific effects under salinity in *Oryza* species.

The splicing event involving the 5′ UTR in *Oryza* AA genomes does not affect ORF length but may influence transcript stability or translatability. Thus, intron inclusion in promoters of maize *Adh1*, *Sh1*, *Bx1*, or *Act* or 5′ UTRs (naturally occurring) of *A. thaliana* polyubiquitin and *EF1-a* genes enhances reporter gene expression several fold (Liu et al., 2001). 5′ UTR variants of *AtZIF2* and *AtPSY* (phytoene synthase) resulting from alternative splicing show distinct translation efficiencies that result in differential zinc tolerance and carotogenesis respectively (Remy et al., 2014; Álvarez et al., 2016).

In plants, stress-induced retention of the last intron is a widely recognized phenomenon (Ner-Gaon et al., 2004). The intron retaining NHX1 (i13) transcript seems to be highly expressed in leaf tissues of most *Oryza* species (Supplementary Figure S9). Retention of the 13th intron would result in PTC of *Oryza* NHX1 and potentially target the transcript for NMD. However, increasing evidence suggests that intron-retaining intermediate transcripts in plants may be preferentially sequestered to escape NMD. This sequestration is reversed under specific environmental cues, triggering intron removal by the spliceosomal machinery and allowing for productive translation and is a mechanism to allow rapid protein synthesis in the absence of transcription (Boothby et al., 2013; Gohring et al., 2014; Brown et al., 2015; Filichkin et al., 2015). In poplar, there appears to exist an inverse relationship between the fully spliced and differential intron retention-harboring mRNA isoforms under abiotic stress conditions (Filichkin et al., 2018). Thus, the *OcNHX1* correctly spliced cDNA and the 13th intron retaining isoform *OcNHX1i13*, show opposite regulation in tissues under salinity and also in leaf tissues of most *Oryza* species under salinity, suggesting a similar mechanism may also be operative in *Oryza NHX1* transcripts.

The 11-bp insertion in the *rOcNHX1* retrocopy sequence leads to premature ORF truncation. *rOcNHX1* has the potential to code for a long non-coding RNA (lncRNA). Retrogenes have been reported from rice, *Populus trichocarpa* and *Arabidopsis* (Zhang et al., 2005; Zhu et al., 2009; Sakai et al., 2011). Tissue specificity of retrogenes in rice is similar to their source genes with expression between retrocopies and source genes correlating across tissues (Sakai et al., 2011). *rOcNHX1* too, appears to show a tissue specific expression profile that is similar to the correctly spliced transcript under salinity in *O. coarctata*. *rOcNHX1* adds one more layer of complexity in the post-transcriptional regulation of *NHX1* in *O. coarctata*.

## Data Availability Statement

The raw data supporting the conclusions of this article will be made available by the authors, without undue reservation, to any qualified researcher.

## Author Contributions

GS and VJ carried out the bulk of this study. GS carried out the qRT-PCR. PP and RR contributed to RT-PCR analysis, cloning, and sequencing. RSS carried out bioinformatic analysis. RS, LS, KR, Z-HC, MZ, SS, and GV contributed to overall planning and design. GV and GS wrote the manuscript. All authors have read and edited the final manuscript.

## Conflict of Interest

The authors declare that the research was conducted in the absence of any commercial or financial relationships that could be construed as a potential conflict of interest.

## Footnotes

1 http://plants.ensembl.org/index.html

2 https://www.ebi.ac.uk/Tools/hmmer/

3 https://www.ebi.ac.uk/Tools/msa/clustalw2/

## Abbreviations

AS: alternative splicing
CPA: cation/proton antiporters
DCR: distal conserved region
IR: intron-retention
MSU: Michigan State University
NHX: Na^+^(K^+^)/H^+^ exchanger
NMD: nonsense mediated decay
ORF: Open reading Frame
PTC: premature termination codons
UTR: Un-translated Region.
