# Supplementary material for: Distinct Evolutionary Origins of Intron Retention Splicing Events in NHX1 Antiporter Transcripts Relate to Sequence Specific Distinctions in Oryza Species
Source: Front Plant Sci. 2020 Mar 11;11:267. doi: 10.3389/fpls.2020.00267 (PMC7078337; doi:10.3389/fpls.2020.00267)
Supplement: Supplementary file 1 [file Table_1.DOCX]

**Supplementary Table 1. NHX1 antiporter genomic, coding (cds), and protein (aa) lengths from *Oryza* spp. based on data retrieved from Ensembl Plants** (<http://plants.ensembl.org/index.html>) **and *Oryza sativa* subsp. *japonica* (IRGSP-1.0; NCBI: gi|996703426|ref|NC_029262.1).**

| ***Oryza.* spp.** | **Genome type** | ***Genomic DNA (bp)*** | ***cds (bp)*** | ***Protein (aa)*** |
| --- | --- | --- | --- | --- |
| *O. coarctata* | KKLL | 4103 | 1620 | 540 |
| *O. brachyantha* | FF | 4535 | 1614 | 538 |
| *O. punctata* | BB | 4165 | 1611 | 537 |
| *O. longistaminata* | AA | 4472 | 1515 | 505 |
| *O. meridionalis* | AA | 4149 | 1611 | 537 |
| *O. rufipogon* | AA | 4138 | 1608 | 536 |
| *O. nivara* | AA | 4125 | 1608 | 536 |
| *O. glaberrima* | AA | 4139 | 1608 | 536 |
| *O. barthii* | AA | 4137 | 1608 | 536 |
| *O. glumaepatula* | AA | 4124 | 1542 | 513 |
| *O. sativa (j)* | AA | 4138 | 1608 | 536 |
