# Supplementary material for: Distinct Evolutionary Origins of Intron Retention Splicing Events in NHX1 Antiporter Transcripts Relate to Sequence Specific Distinctions in Oryza Species
Source: Front Plant Sci. 2020 Mar 11;11:267. doi: 10.3389/fpls.2020.00267 (PMC7078337; doi:10.3389/fpls.2020.00267)
Supplement: Supplementary file 2 [file Table_2.DOCX]

**Supplementary Table 2: List of primers used in this study.**

| **Sl. No** | **Technique** | **PCR** | **Primer name** | **Primer sequence (5′ to 3′)** | **Size (bp)** |
| --- | --- | --- | --- | --- | --- |
| ***rOcNHX1* genome walking** | | | | | |
| A | Genomic PCR |  | Seq Fwd | CCTGGCAAATCCATTGGGAT | 1001 |
|  |  |  | OcNHX UTR Rev | TGTGTAAAATGATTTTGCTTGGTTGAT |  |
| B | Genomic PCR |  | OcNHX1 Exon Fwd 2 | CTTGGAGTATTTGCTGGATTGCT | 583 |
|  |  |  | Seq Rev | CAGTGATGGTGCTGGTGAT |  |
| C | Inverse PCR | 1° PCR | OcNHX Fwd A | CTGAACACGACCTCCGACTACG | 1193 |
|  |  |  | OcNHX1 Exon Rev A | TGGCCAGATCTTGTAAACTTATTGTAAG |  |
|  |  | 2° PCR | OcNHX Fwd A | CTGAACACGACCTCCGACTACG | 881 |
|  |  |  | OcNHX1 Exon Rev 3 | GCAAATGCGTGCTTGGTTGTAAC |  |
|  |  | 3° PCR | OcNHX Fwd A | CTGAACACGACCTCCGACTACG | 499 |
|  |  |  | OcNHX Rev 1 | GCAAATACTCCAAGGAAGGTGC |  |
| D | Nested Genomic PCR | 1° PCR | Retro RT Fwd A | CAGATCTTCCAATCTAGATTCGC | 1100 |
|  |  |  | Seq Rev | CAGTGATGGTGCTGGTGAT |  |
|  |  | 2° PCR | Retro RT Fwd A | CAGATCTTCCAATCTAGATTCGC | 563 |
|  |  |  | OcNHX1 Exon Rev 4 | CCTGGTTGAGGACCTGCAATGT |  |
|  |  | 3° PCR | Retro RT Fwd A | CAGATCTTCCAATCTAGATTCGC | 344 |
|  |  |  | OcNHX Rev P | GTCCCAACAGCTCCAAATAATGTGAT |  |
| E | Nested Genomic PCR | 1° PCR | OcNHX Fwd 2 | GAGACATGTAAAACGTGACAG | 1631 |
|  |  |  | OcNHX1 Rev 2 | CGAACTTGCGCCAGTAGTAGTG |  |
|  |  | 2° PCR | OcNHX Fwd 2 | GAGACATGTAAAACGTGACAG | 1040 |
|  |  |  | OcNHX Exon Rev 3 | GCAAATGCGTGCTTGGTTGTAAC |  |
|  |  | 3° PCR | OcNHX Fwd 2 | GAGACATGTAAAACGTGACAG | 631 |
|  |  |  | OcNHX Exon Rev 4 | CCTGGTTGAGGACCTGCAATGT |  |
|  | | | | | |
| **Primer name** | | | | **Primer sequence (5′ to 3′)** | |
| **Isolation of splice variants** | | | | | |
| OcNHX1 Ex-jn Fwd | | | | GCTTACAATAAGTTTACAAGATCTG | |
| OcNHX Ex Rev1 | | | | CTTGTCAGGAGAGGAGAATTCA | |
| OcNHX1 Ex13 Fwd | | | | ATCACCAGCACCATCACTG | |
| OcNHX Ex Rev1 | | | | CTTGTCAGGAGAGGAGAATTCA | |
| OcNHX1 Ex-In Fwd1 | | | | AGCACTATGGTGAGTCATCTTG | |
| OcNHX Ex Rev1 | | | | CTTGTCAGGAGAGGAGAATTCA | |
| OcNHX1 Ex-jn Fwd | | | | GCTTACAATAAGTTTACAAGATCTG | |
| OcNHX1 In-Ex Rev1 | | | | CATCATCCCAATCACCCTAATAAAC | |
| **qRT-PCR analysis** | | | | | |
| OcActin Fwd | | | | GAAAGGAAGTACAGTGTCTGGATTG | |
| OcActin Rev1 | | | | AAGCATTTCCTGTGCACAATGGAT | |
| OcNHX1 RT Fwd2 (RT-F2) | | | | GAGAGGAGCTGTGTCGATTGC | |
| OsNHX1 RT Rev2 (RT-R2) | | | | GGTAGCAGCAGCCTGATCAATG | |
| OsNHX1 Ex-In jn Fwd2 (i13F) | | | | AGCACTATGGTGAGTCATCTTACTGC | |
| OcNHX1 Intron Rev1 (i13R) | | | | GCTTATGATCAAGTGATTGGATGC | |
| OsNHX1 Fwd1 | | | | GAGTTTTGTAGCGAGCTCGCGC | |
| OsNHX1 Rev1 | | | | CTCACCGGCGGCGACTTCTC | |
| OsNHX1 5’ URT Fwd1 | | | | TGCGAAGCCAACCGAGAGA | |
| OsNHX1 5’ URT Fwd2 (UTR-F2) | | | | GCCAACCGAGAGAGGTCTCGATAC | |
| OsNHX1 5’UTR-Sp Fwd (Sp-F1) | | | | GCCAACCGAGAGAGGCATTCACCA | |
| OsNHX1 5’UTR Rev2 (UTR-R2) | | | | GCGGAGCTTTAACCAACTAATCCA | |
| **3' RACE amplification** | | | | | |
| GSP1 | | | | AGCACTATGGTGAGTCATCTTG**3** | |
| GSP2 | | | | AGCCCTCAACATTAAGCTAACC**3’** | |
