# Supplementary figures and images for: Distinct Evolutionary Origins of Intron Retention Splicing Events in NHX1 Antiporter Transcripts Relate to Sequence Specific Distinctions in Oryza Species
Source: Front Plant Sci. 2020 Mar 11;11:267. doi: 10.3389/fpls.2020.00267 (PMC7078337; doi:10.3389/fpls.2020.00267)

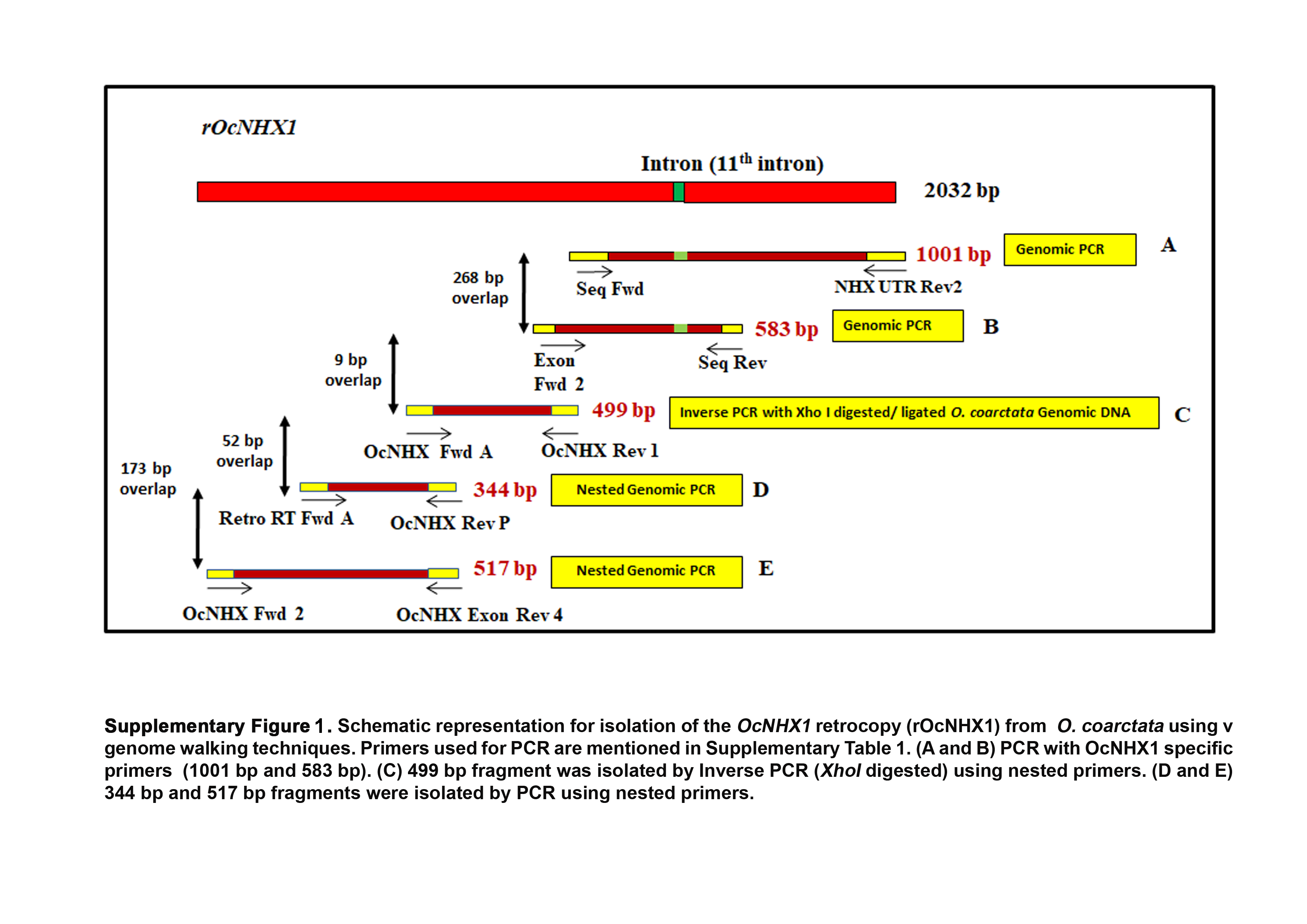

Supplement: Supplementary file 3 [file Image_1.TIF]

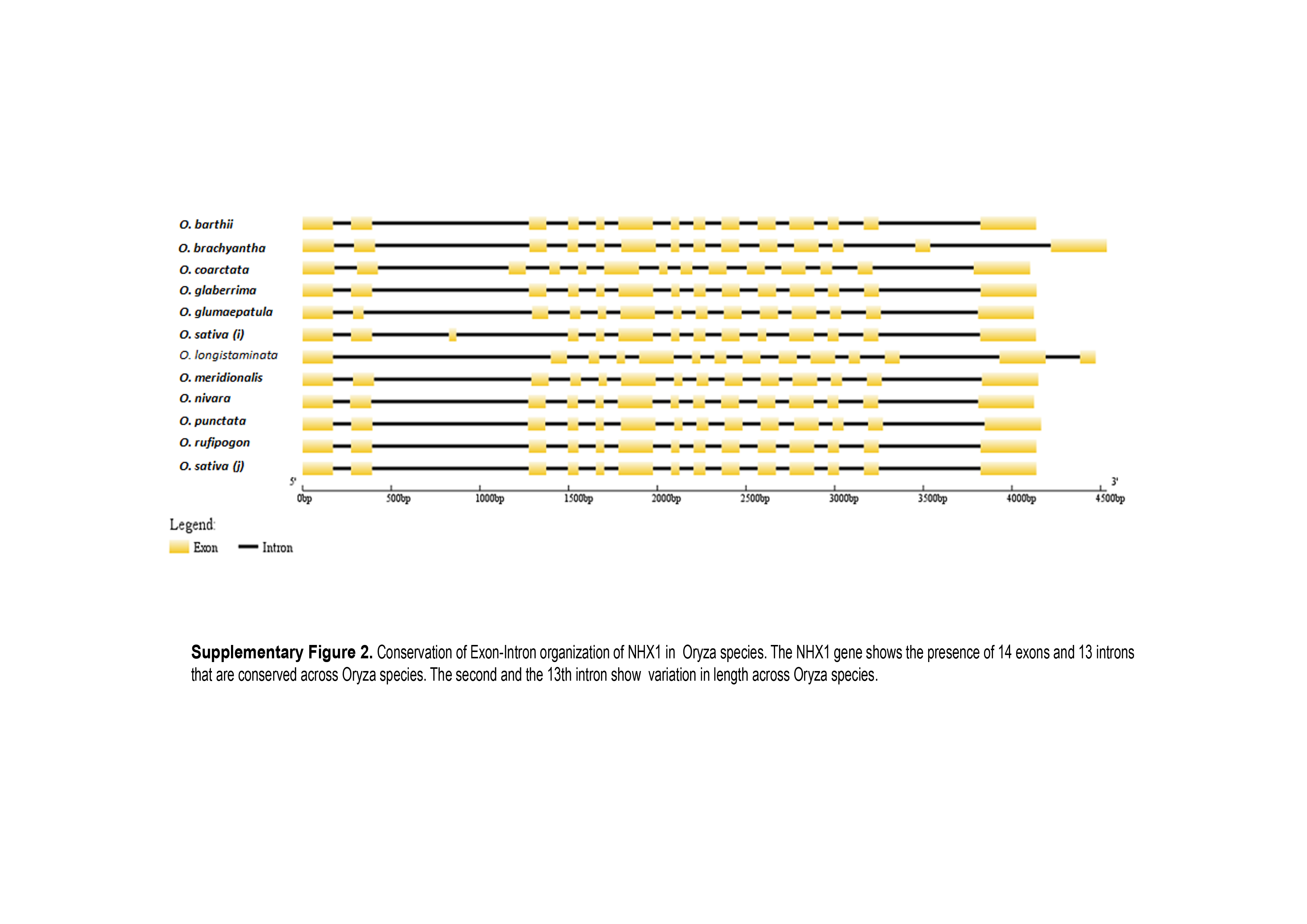

Supplement: Supplementary file 4 [file Image_2.TIF]

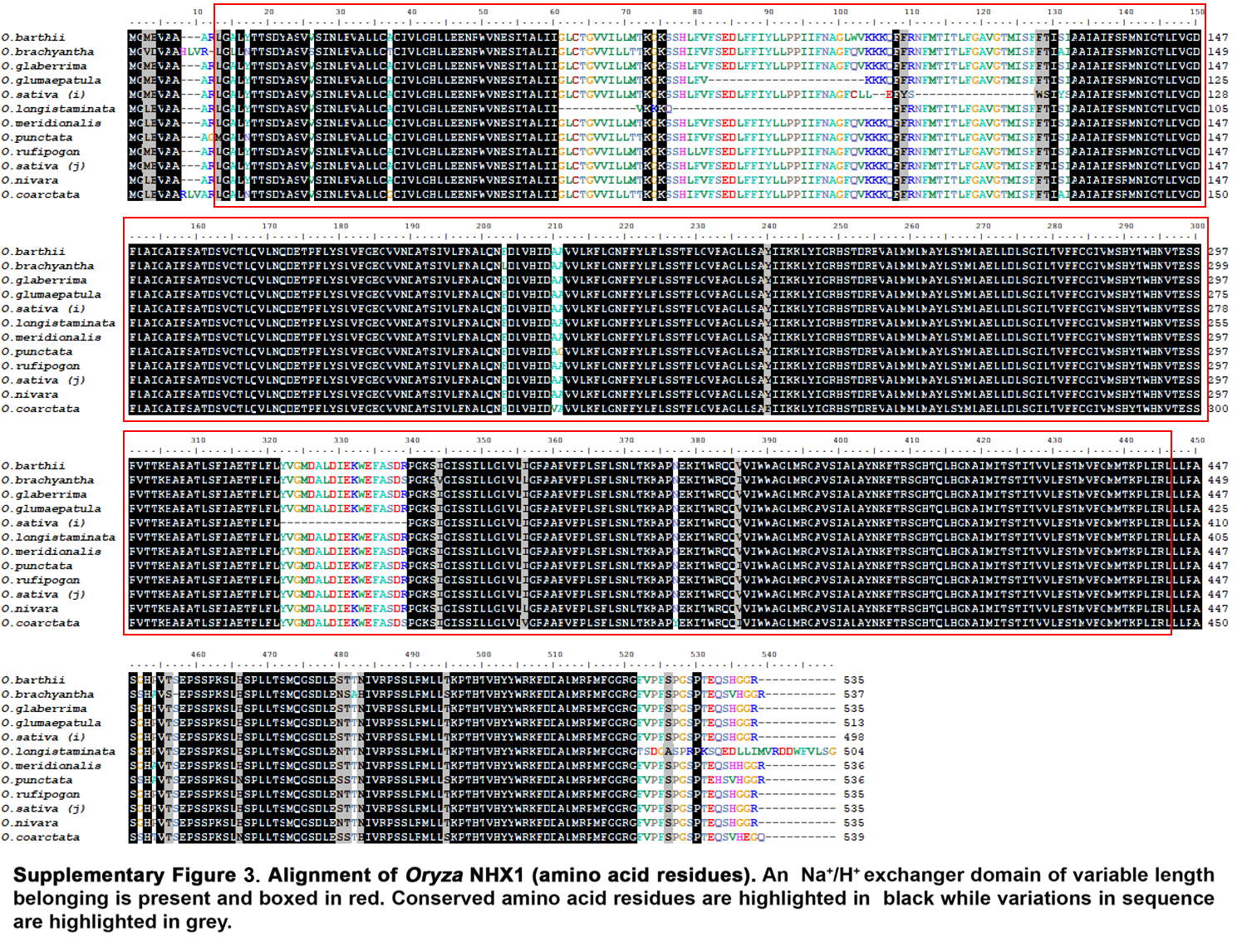

Supplement: Supplementary file 5 [file Image_3.TIF]

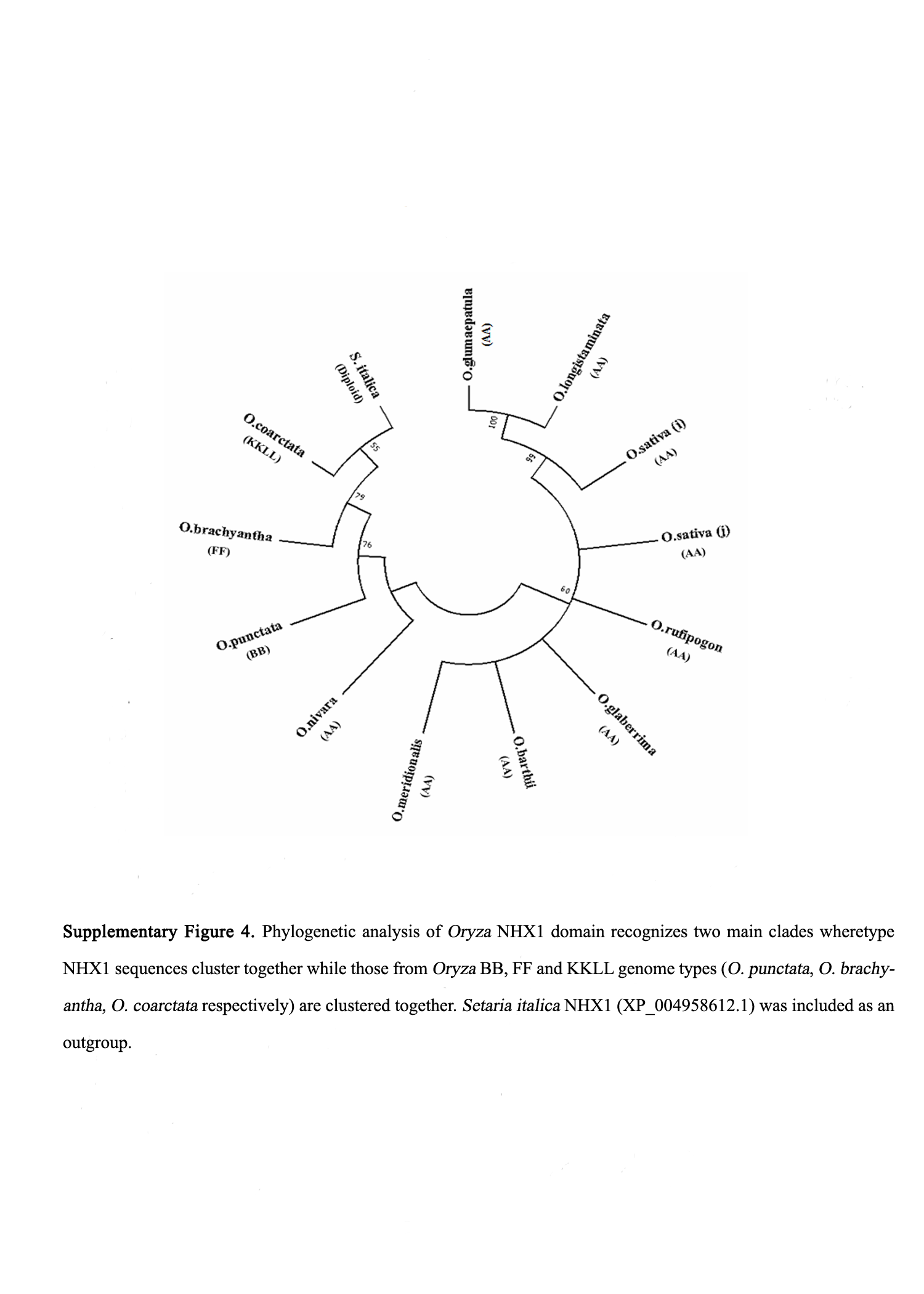

Supplement: Supplementary file 6 [file Image_4.TIF]

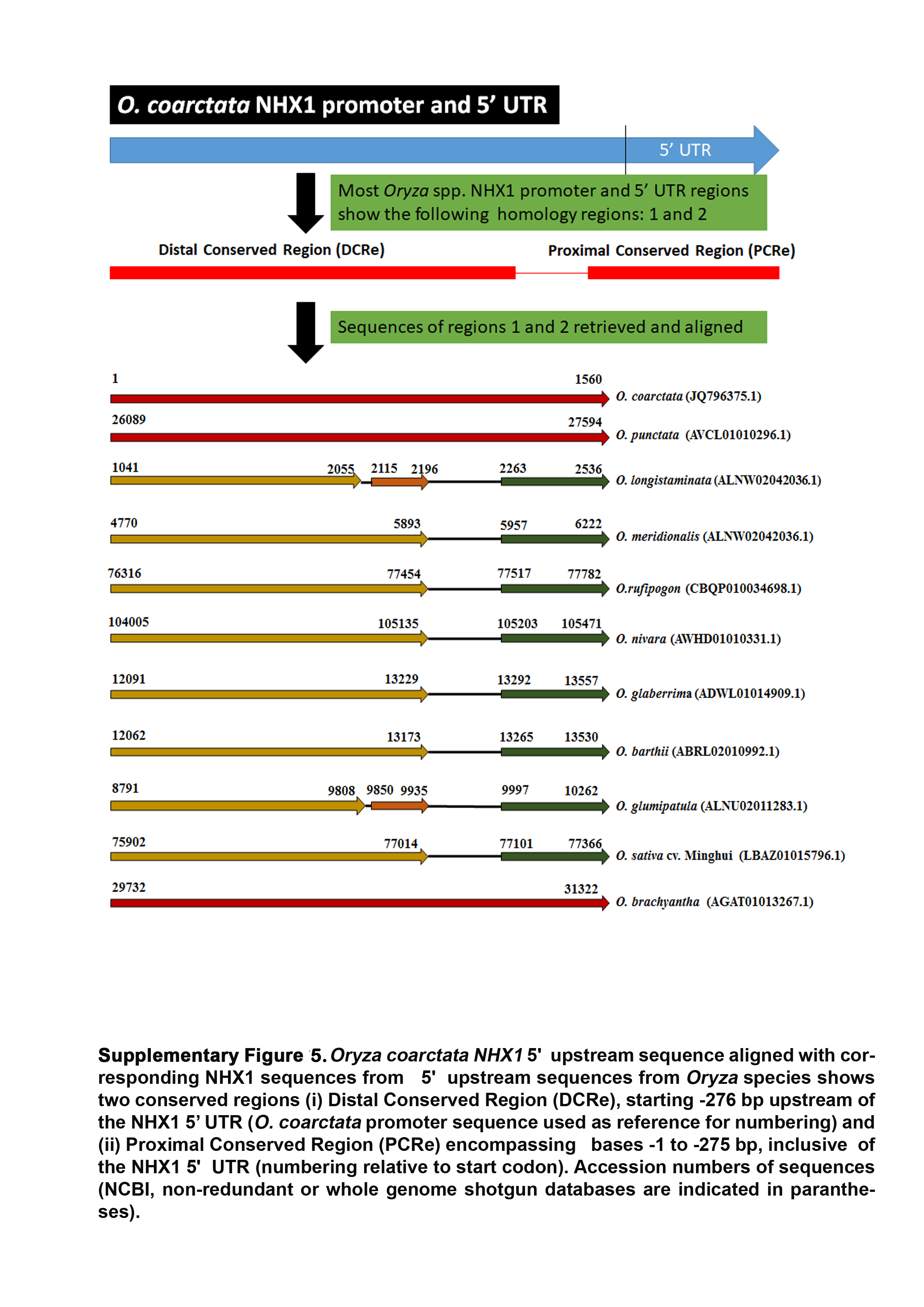

Supplement: Supplementary file 7 [file Image_5.TIF]

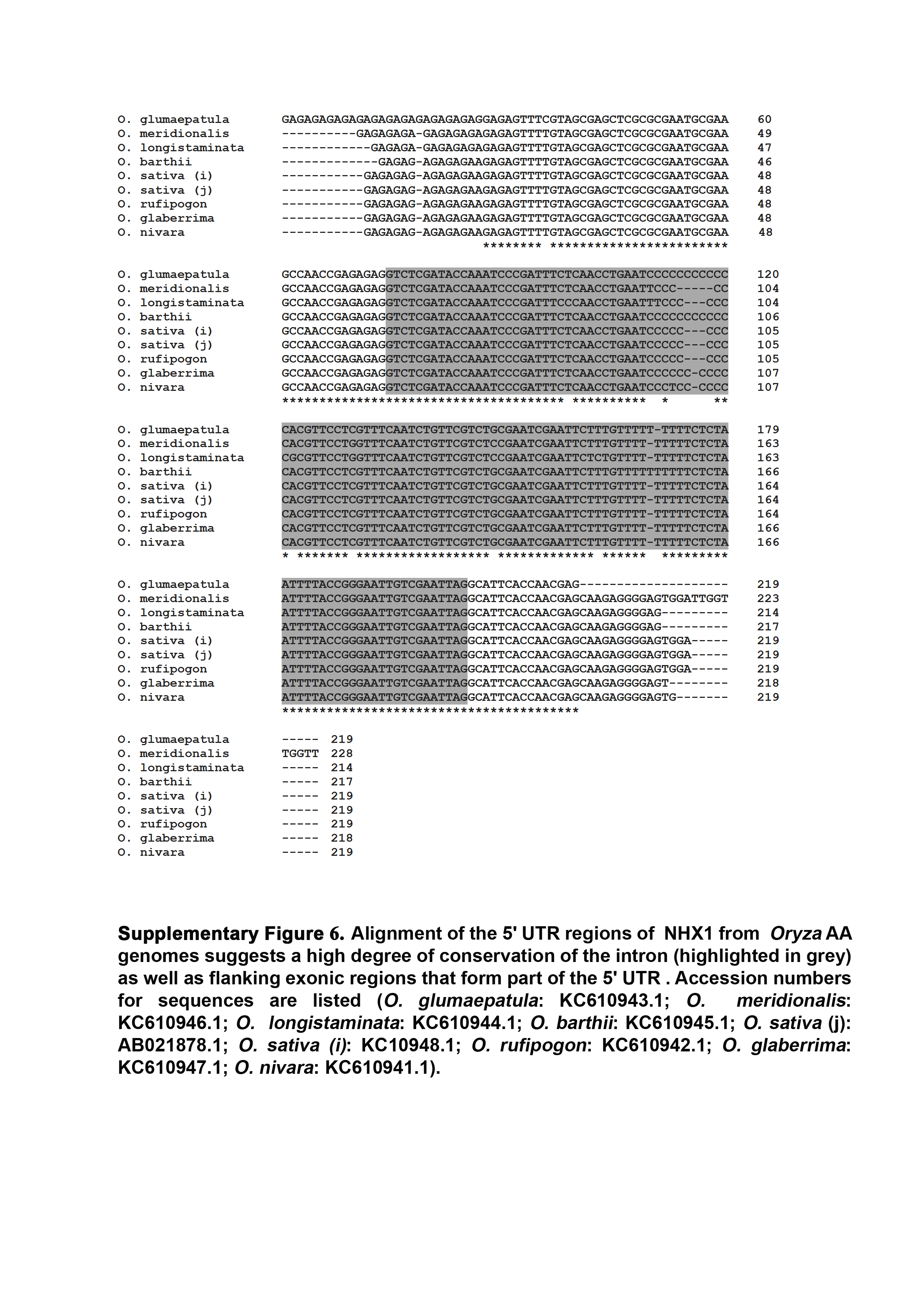

Supplement: Supplementary file 8 [file Image_6.TIF]

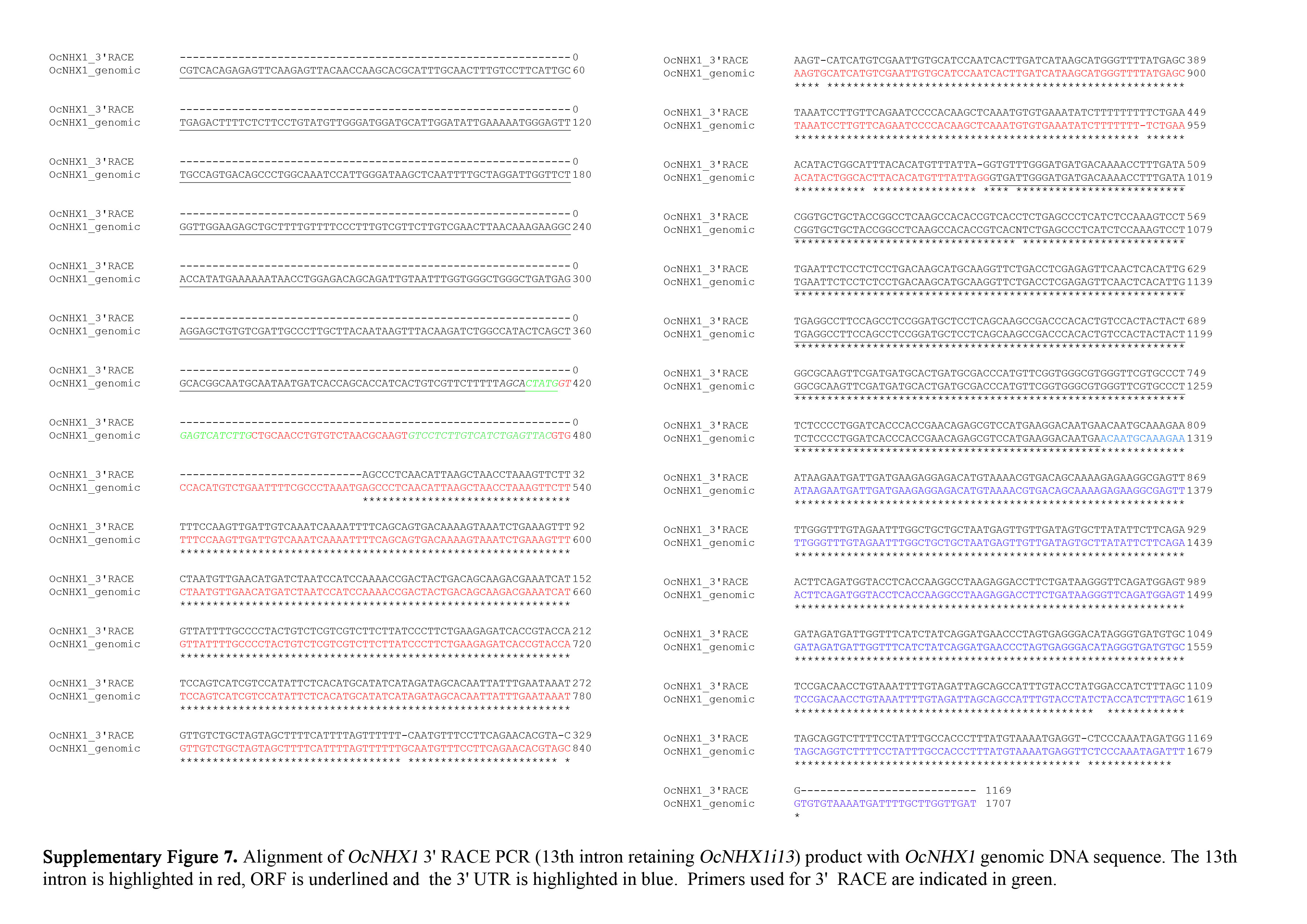

Supplement: Supplementary file 9 [file Image_7.TIF]

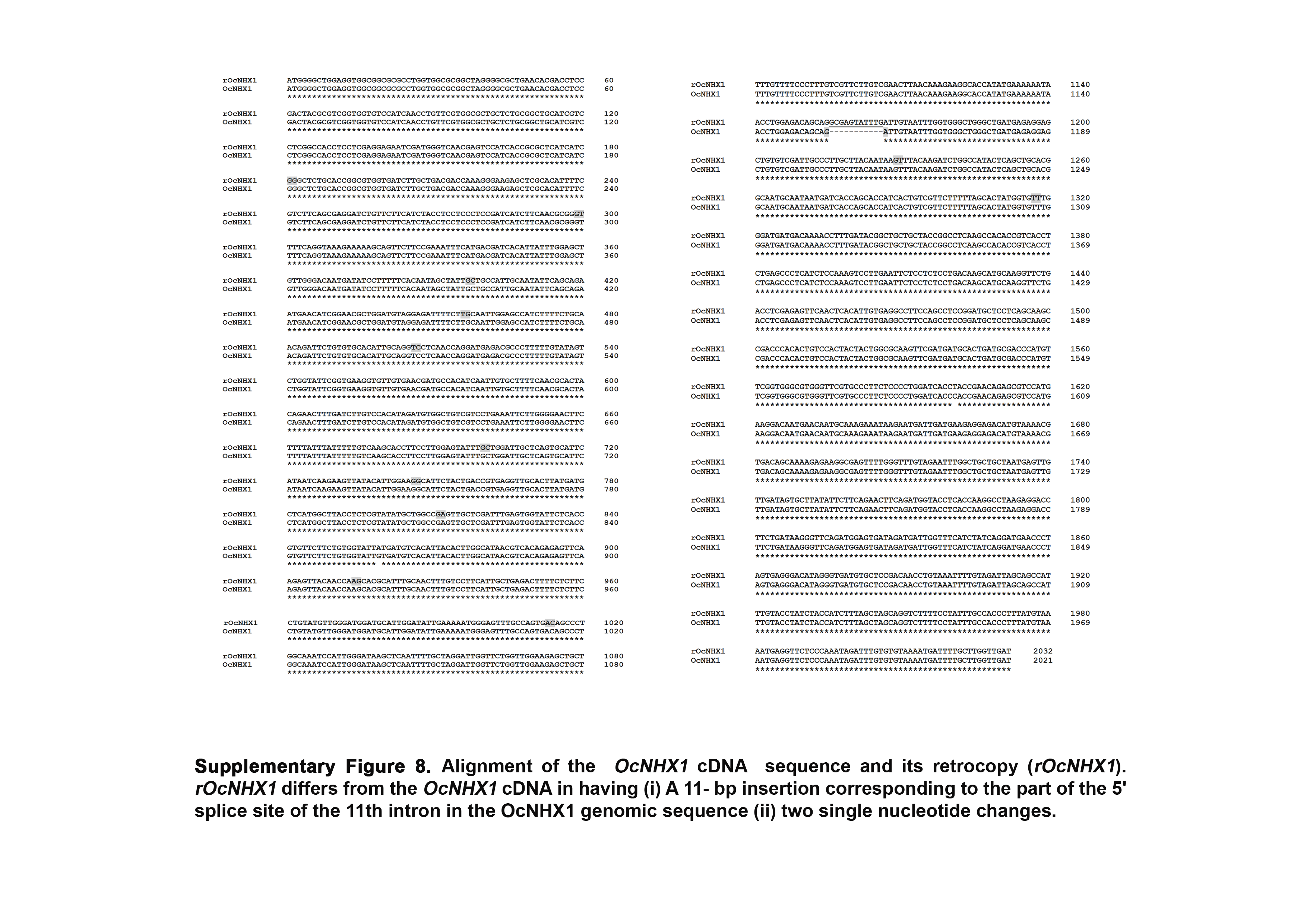

Supplement: Supplementary file 10 [file Image_8.TIF]

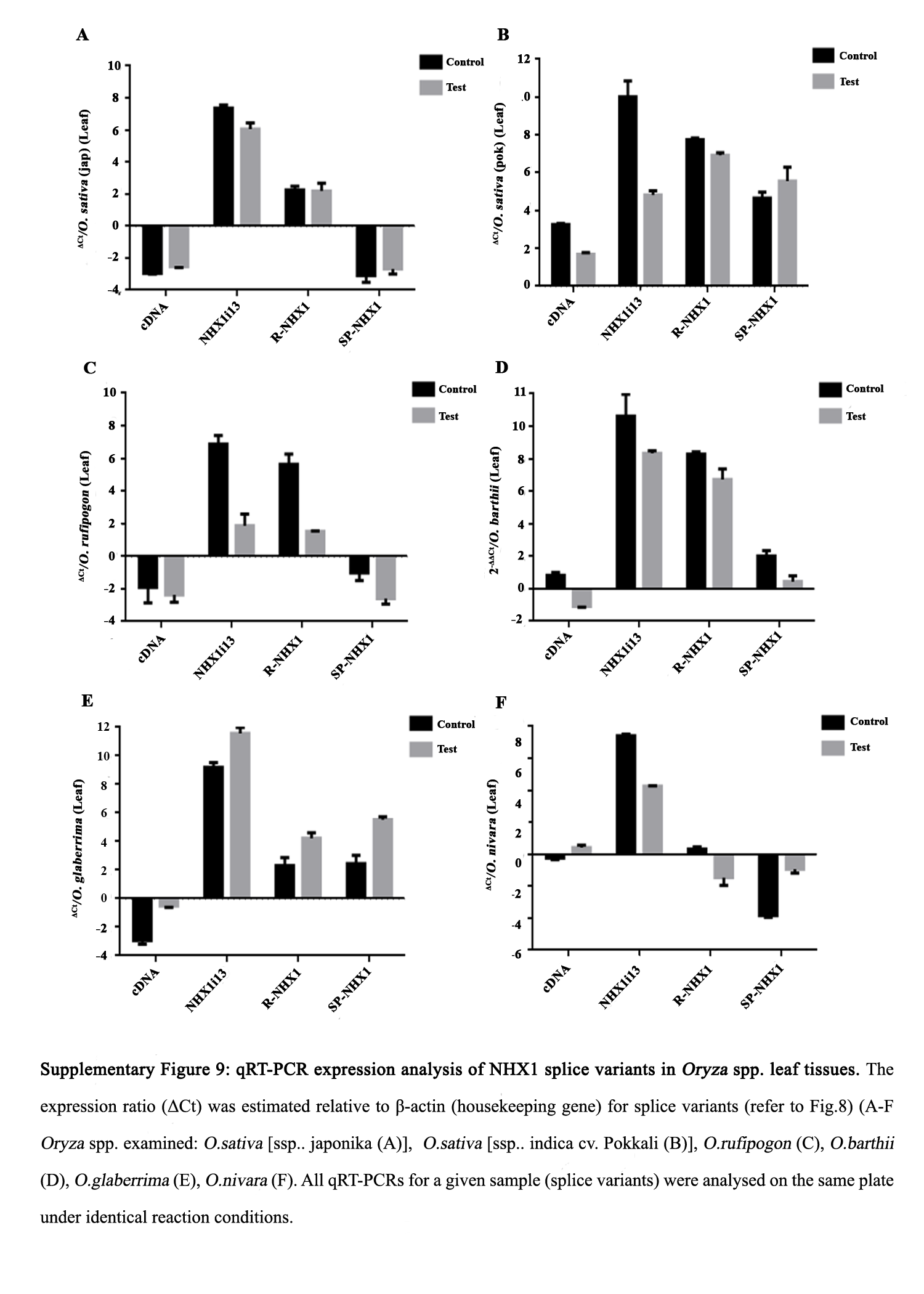

Supplement: Supplementary file 11 [file Image_9.TIF]

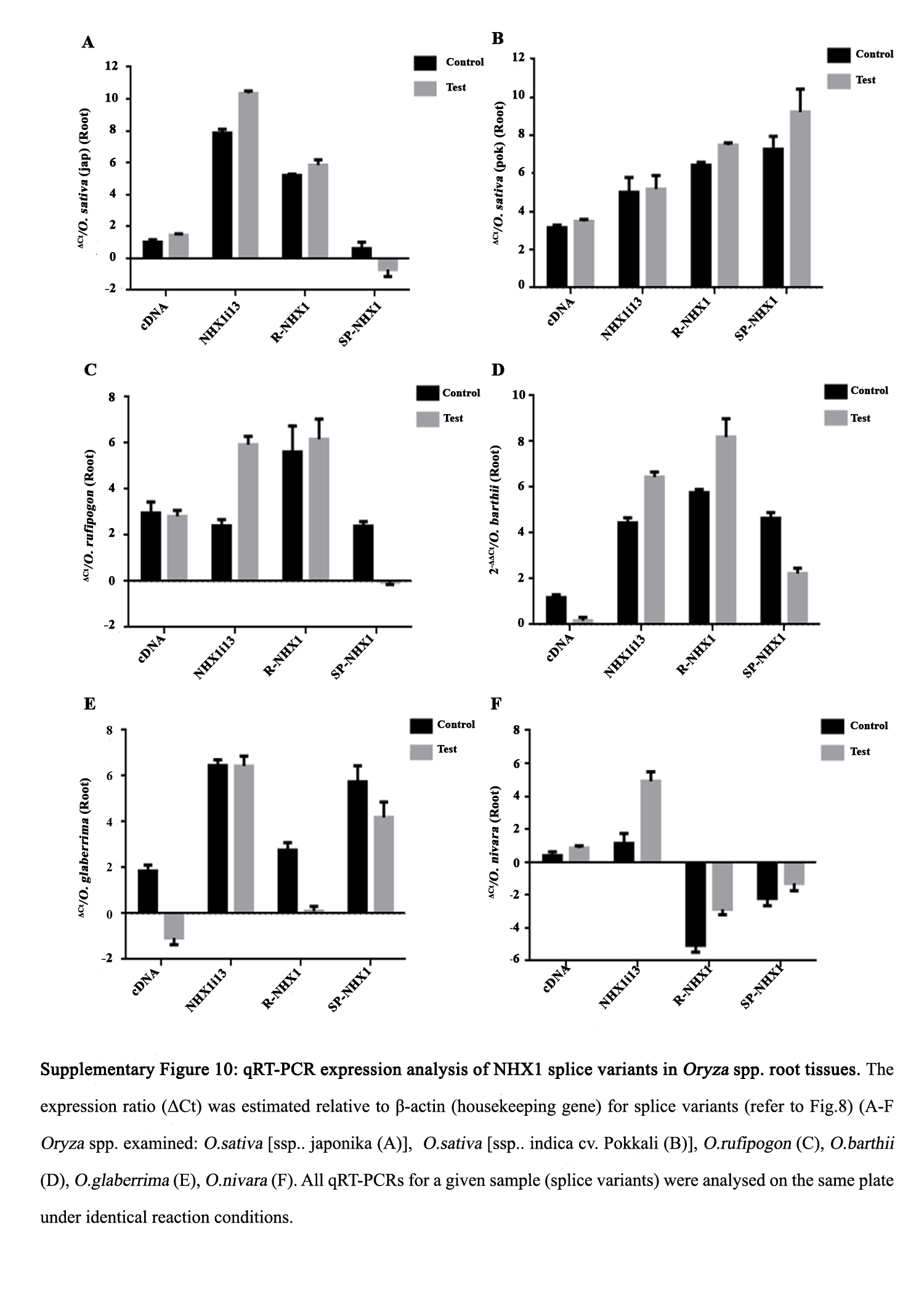

Supplement: Supplementary file 12 [file Image_10.TIF]

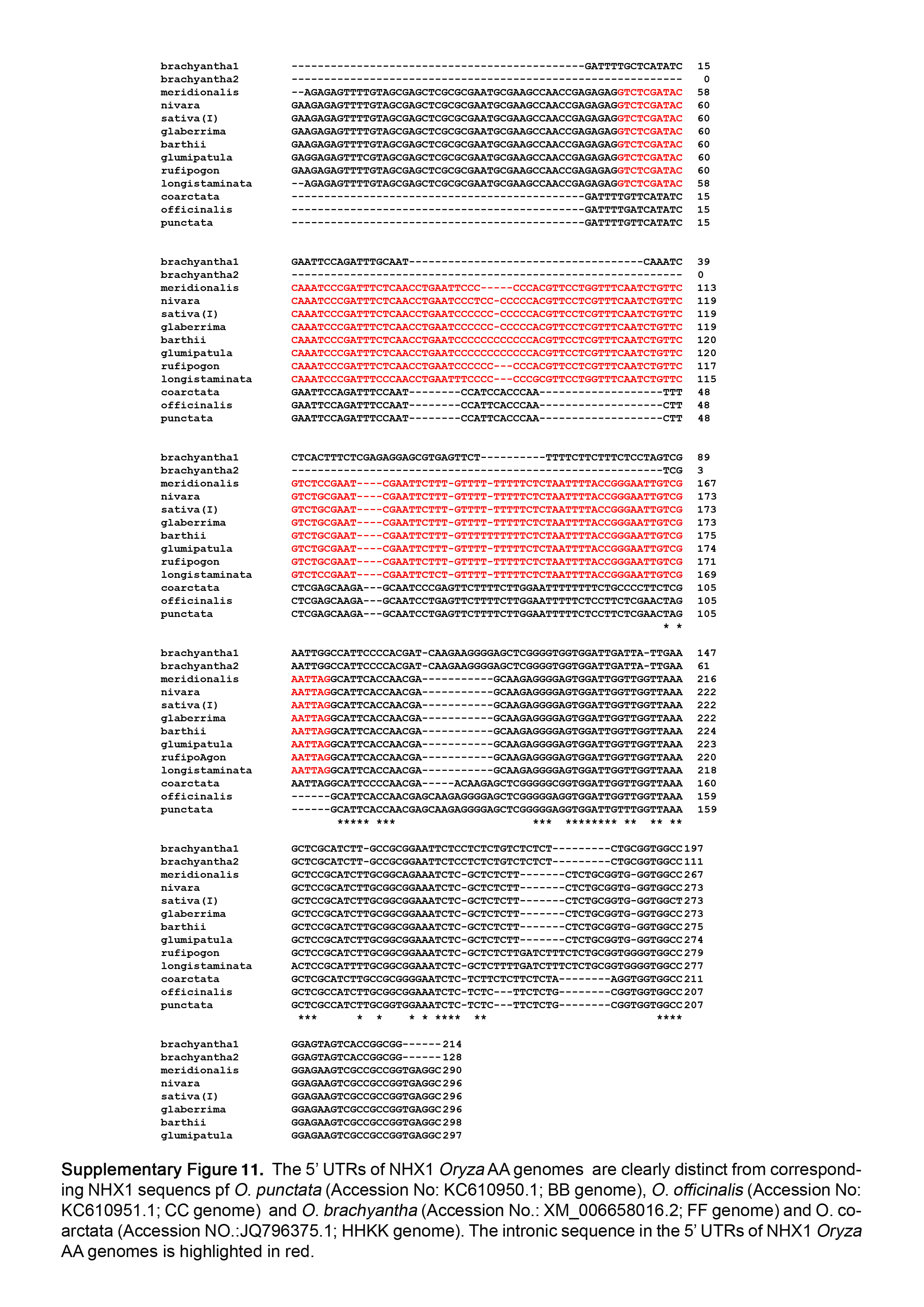

Supplement: Supplementary file 13 [file Image_11.TIF]
